# Eukaryotic translation initiation factor 5A and its posttranslational modifications play an important role in proliferation and potentially in differentiation of the human enteric protozoan parasite *Entamoeba histolytica*

**DOI:** 10.1371/journal.ppat.1008909

**Published:** 2021-02-16

**Authors:** Ghulam Jeelani, Tomoyoshi Nozaki

**Affiliations:** Department of Biomedical Chemistry, Graduate School of Medicine, The University of Tokyo, Japan; University of Virginia, UNITED STATES

## Abstract

The eukaryotic translation initiation factor 5A (eIF5A) is a highly conserved protein and is essential in all eukaryotes. However, the specific roles of eIF5A in translation and in other biological processes remain elusive. In the present study, we described the role of eIF5A, its posttranslational modifications (PTM), and the biosynthetic pathway needed for the PTM in *Entamoeba histolytica*, the protozoan parasite responsible for amoebic dysentery and liver abscess in humans. *E*. *histolytica* encodes two isotypes of eIF5A and two isotypes of enzymes, deoxyhypusine synthase (DHS), responsible for their PTM. Both of the two eIF5A isotypes are functional, whereas only one DHS (EhDHS1, but not EhDHS2), is catalytically active. The DHS activity increased ~2000-fold when EhDHS1 was co-expressed with EhDHS2 in *Escherichia coli*, suggesting that the formation of a heteromeric complex is needed for full enzymatic activity. Both *EhDHS1* and *2* genes were required for *in vitro* growth of *E*. *histolytica* trophozoites, indicated by small antisense RNA-mediated gene silencing. In trophozoites, only *eIF5A2*, but not *eIF5A1*, gene was actively transcribed. Gene silencing of *eIF5A2* caused compensatory induction of expression of *eIF5A1* gene, suggesting interchangeable role of the two eIF5A isotypes and also reinforcing the importance of eIF5As for parasite proliferation and survival. Furthermore, using a sibling species, *Entamoeba invadens*, we found that *eIF5A1* gene was upregulated during excystation, while *eIF5A2* was downregulated, suggesting that *eIF5A1* gene plays an important role during differentiation. Taken together, these results have underscored the essentiality of eIF5A and DHS, for proliferation and potentially in the differentiation of this parasite, and suggest that the hypusination associated pathway represents a novel rational target for drug development against amebiasis.

## Author summary

Eukaryotic initiation factor 5A is a ubiquitous protein that is essential for cell proliferation. We examined the maturation, regulation, and function of eIF5A in *E*. *histolytica*. We found by small antisense RNA-mediated gene silencing that EhDHS1/2 and EheIF5A2 are essential for growth of *E*. *histolytica* trophozoites. We further found that only one of two isotypes of eIF5A, EheIF5A2, was constitutively expressed in the trophozoite stage and silencing of *EheIF5A2* gene caused overexpression of the other eIF5A isotype (EheIF5A1) to partially rescue the growth defect in this parasite. Furthermore, we found that transcription of *eIF5A1* gene was stage-specifically upregulated during excystation in *E*. *invadens*. Taken together, we have demonstrated for the first time that the two eIF5As play important and distinct roles in *Entamoeba* biology. This study has also provided an answer to a long-standing conundrum on the biological importance of polyamines: spermidine is needed for eIF5A hypusination, which is essential for protein translation in *Entamoeba*. Our work will provide an insight into the physiological significance of eIF5A and its post-translational modifications in other pathogenic eukaryotes and potentially lead to the formulation of control measures against parasitic diseases.

## Introduction

**Fig 1:**
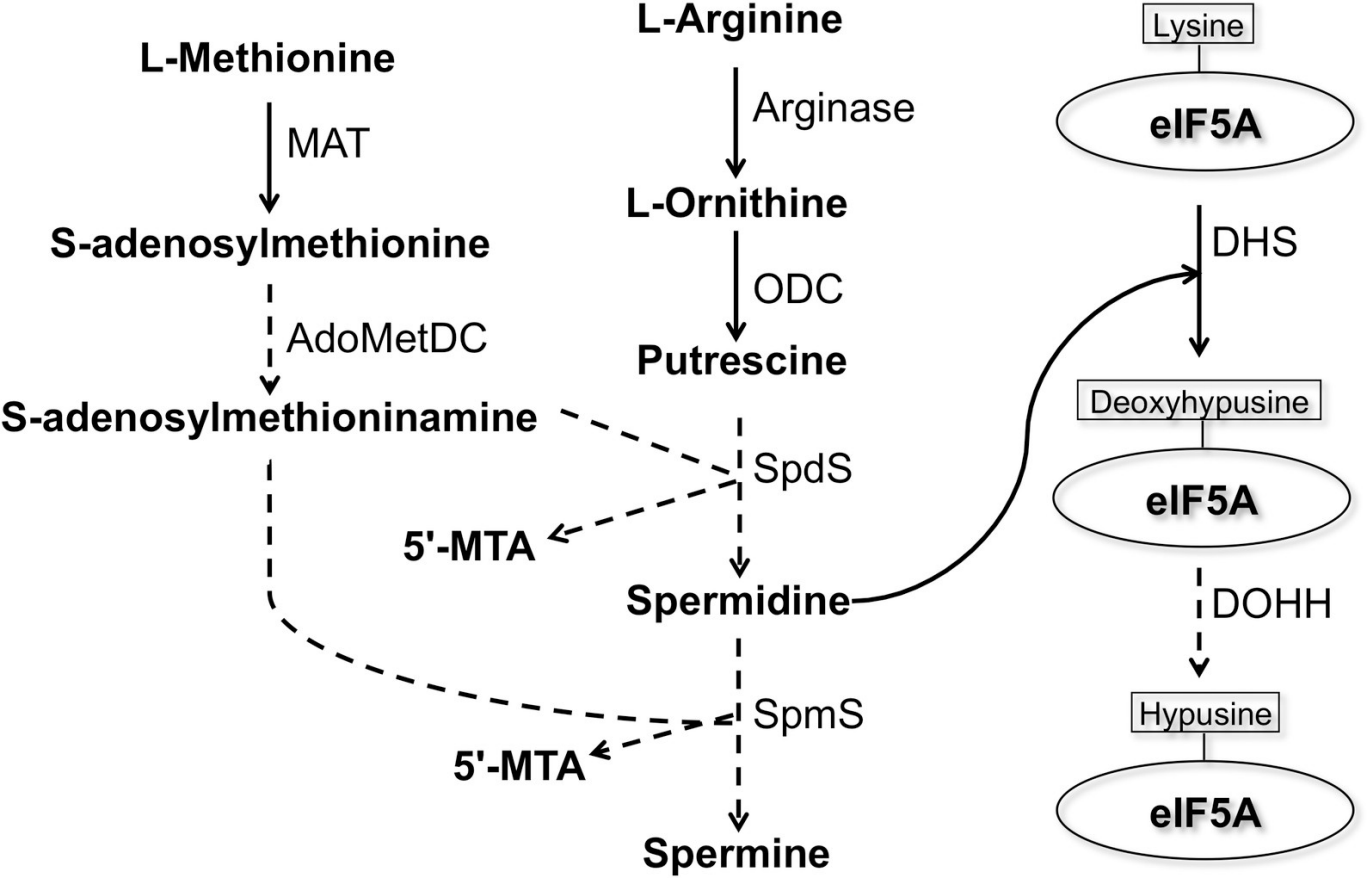
Presumed biosynthetic pathway of polyamines and deoxyhypusine/hypusine modifications on eukaryotic initiation factor 5A in *E*. *histolytica*. Solid lines represent the steps catalyzed by the enzymes whose encoding genes are present in the *E*. *histolytica* genome, whereas dashed lines indicate those absent or not yet identified. Abbreviations: 5’-MTA, 5’-methylthioadenosine; MAT, S-adenosylmethionine synthetase; ODC, ornithine decarboxylase, AdoMetDC, S-adenosylmethionine decarboxylase; SpdS, spermidine synthase; SpmS, spermine synthase; eIF5A, eukaryotic initiation factor 5A; DHS, deoxyhypusine synthase; DOHH, deoxyhypusine hydroxylase.

Polyamines are low-molecular-weight nitrogenous bases that are essential for the regulation of cell growth and development [1]. Due to their polycationic nature, polyamines have the ability to interact electrostatically with the majority of polyanionic macromolecules in cells and thereby influence a variety of processes including cell differentiation and proliferation, embryonic development, and apoptosis [2]. As a consequence, increased concentrations of polyamines and their biosynthetic enzymes are observed in highly proliferating cells such as cancerous cells and parasitic organisms [3]. One of the demonstrated universal roles of polyamines, particularly spermidine, in eukaryotic cells is the formation of hypusine on the eukaryotic initiation factor 5A (eIF5A) (Fig 1). Hypusine is an unusual amino acid [N (ε)- (4-amino-2-hydroxybutyl)-lysine], which is uniquely synthesized on eIF5A at a specific lysine residue from spermidine by two catalytic steps [4]. This post-translational modification (PTM) in eukaryotes is achieved by the sequential reactions catalyzed by two enzymes: deoxyhypusine synthase (DHS) and deoxyhypusine hydroxylase (DOHH) (Fig 1) [4]. Hypusination is the most specific PTM known to date [5] and it is essential for eIF5A activity [6]. eIF5A is involved in elongation [7], termination [8], and stimulation of peptide bond formation [9], and it facilitates protein synthesis by resolving polyproline-induced ribosomal stalling; thus, its role seems indispensable in synthesis of proline repeat-rich proteins [10].

*E*. *histolytica* is a unicellular parasitic protozoan responsible for human amebiasis. The World Health Organization estimates that approximately 50 million people worldwide suffer from invasive amebic infections, resulting in 40 to 100 thousand deaths annually [11]. As a parasite, *E*. *histolytica* needs to be able to cope with a wide variety of environmental stresses, such as fluctuations in glucose concentration, changes in pH, pO_2_, temperature, and host immune responses including oxidative and nitrosative species from neutrophils and macrophages during the life cycle [12]. Our previous metabolomic analyses have indicated that polyamines including putrescine, spermidine, and spermine are abundantly present in the proliferating and disease-causing trophozoites and the levels of these metabolites dramatically decrease during stage conversion from trophozoites to dormant cysts [13]. Interestingly, genome-wide survey of the reference genome (http://amoebadb.org/amoeba/) suggested that *E*. *histolytica* lacks a few key enzymes involved in polyamine biosynthesis, conserved in other bacterial and eukaryotic organisms: *S*-adenosylmethionine decarboxylase (AdoMetDC), spermine synthase (SpmS), and spermidine synthase (SpdS), suggesting that *E*. *histolytica* may possess a unique pathway or enzymes for polyamine biosynthesis, which can be further explored as a drug target against amebiasis.

It has been reported that spermidine is needed to hypusinate the translation factor eIF5A [14]. The *E*. *histolytica* genome revealed that this parasite possesses genes for deoxyhypusine synthase and eIF5A; however, *E*. *histolytica* seems to lack DOHH, which is involved in the formation of mature hypusinated eIF5A (Fig 1). DHS has also been shown to be essential in all species where it was studied including mammals, yeast [15], and the kinetoplastids, *Trypanosoma brucei* [16] and *Leishmania* [17]. While most eukaryotes possess only a single *DHS* gene, *Entamoeba*, *Leishmania*, *and Trypanosoma* possess more than one *DHS* or *DHS-like* genes [18]. The presence of two *DHS* genes in these parasitic protozoa may be suggestive of unknown biological significance.

In the present study, we demonstrate that only one of the two *EhDHS* genes from *Entamoeba* encodes for the enzymatically active DHS. We show that the other DHS isotype encoded by the second gene is needed for the formation of a protein complex, required for maximal enzyme activity. We also show that both *EhDHS1* and *2* genes are required for optimal *in vitro* growth of *E*. *histolytica* cells. We have also found that only eIF5A2 protein, but not eIF5A1, is constitutively expressed in trophozoites and that silencing of *eIF5A2* gene causes compensatory expression of eIF5A1, suggesting that these proteins play partially interchangeable roles for growth or survival. Furthermore, we found that transcription of *eIF5A1* gene is upregulated during excystation while that of *eIF5A2* is downregulated, suggesting that eIF5A1 may play an important role in differentiation. To date, this study represents the first case indicating that eIF5A plays an important role in proliferation and potentially in differentiation of this pathogenic eukaryote.

## Results

### Identification and features of *DHS* and *eIF5A* genes and its encoded proteins from *E*. *histolytica*

**Fig 2:**
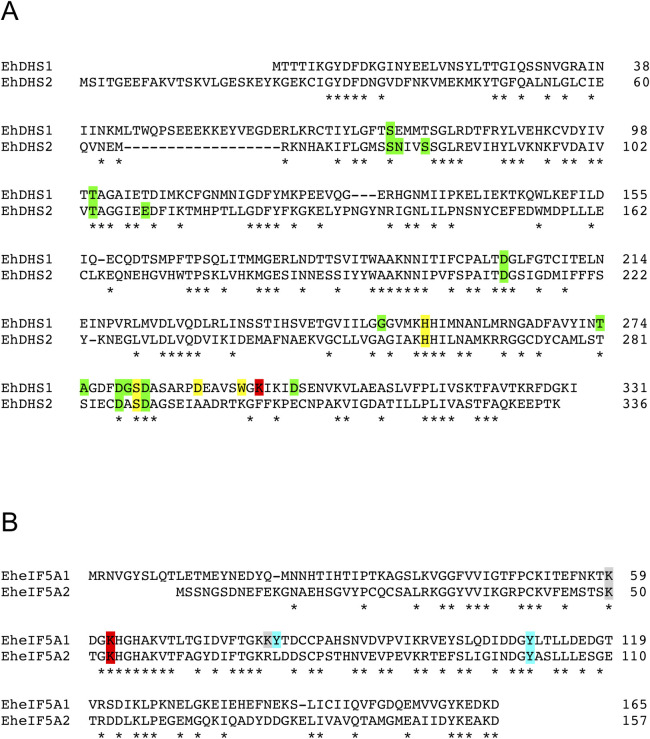
Multiple alignment of *E*. *histolytica* DHS and eIF5A protein sequences. Conserved residues are marked by asterisks (*). Sequence alignment was performed using ClustalW. **(A)** Amino acid sequence alignment of *E*. *histolytica* DHS isoforms. Accession numbers of these sequences are as follows: EhDHS1 (XP_653614), EhDHS2 (XP_653426). The catalytic lysine residue is shown in red background, whereas NAD^+^ and spermidine binding sites are shown in green and yellow background, respectively. **(B)** Amino acid sequence alignment of *E*. *histolytica* eIF5A isoforms. Accession numbers of these sequences are as follows: EheIF5A1 (XP_657374); EheIF5A2 (XP_651531). The conserved lysine residues which are expected to be hypusinated are highlighted in red. Residues which are post-transnationally modified by acetylation and sulfation in human eIF5A, are shown in grey and cyan background, respectively.

While polyamines were previously demonstrated in trophozoites [13], their metabolic roles remained elusive. Since hypusination utilizes spermidine as a substrate, we hypothesized that amebic trophozoites use spermidine for the PTM of eIF5A. A genome-wide survey of eukaryotic DHS, which catalyzes the first step of hypusination of eIF5A, in the *E*. *histolytica* reference genome (AmoebaDB, http://amoebadb.org/amoeba/), by BLASTP analysis using human DHS as a query, revealed that *E*. *histolytica* possesses two possible DHS homologs, which showed 34% mutual identity. We designated EHI_098350 as EhDHS1 and EHI_006030 as EhDHS2. EhDHS1 contains the key catalytic lysine residue known to be conserved among orthologs from other eukaryotes, while the residue is not conserved in EhDHS2 (Fig 2A). Both EhDHS1 and EhDHS2 exhibit 47% amino acid sequence identity to human DHS (HsDHS), which is encoded by a single copy gene (S1 Fig). Multiple sequence alignment shows that EhDHS1 contains a complete set of the amino acid residues implicated for spermidine binding (4/4) that are conserved in HsDHS [19], whereas only two of four residues are present in EhDHS2. Nine and ten out of thirteen residues predicted to be involved in NAD-binding in HsDHS are conserved in EhDHS1 and EhDHS2, respectively (S1 Fig).

A genome wide survey identified four eIF5A proteins (EHI_151540, EHI_186480, EHI_177460, and EHI_151810). They are categorized into two groups (EheIF5A1 and EheIF5A2) based on mutual sequence similarity (S2 Fig). We designated representative allelic isotypes as eIF5A1 for EHI_151540 (and EHI_177460) and eIF5A2 for EHI_186480 (and EHI_151810), respectively. Sequence alignment of the two EheIF5A isotypes by ClustalW program (http://clustalw.ddbj.nig.ac.jp/top-e.html) shows that the two glycine residues adjacent to lysine (red) are conserved in EheIF5A (amino acid positions 61 and 64 in eIF5A1; 52 and 55 in eIF5A2) (Fig 2B). This Gly-X-Y-Gly motif was reported to be critical for the formation of β-turn structure and the proper orientation of the deoxyhypusine/hypusine side chain [20]. Lysine residues at positions 62/53 and 59/50 in eIF5A1/2, respectively, are presumed to be the sites for hypusination and acetylation, respectively [21]. In addition, tyrosine residues at positions 81 and 110 in eIF5A1 and at position 101 in eIF5A2 are likely the sites of sulfation [22]. The highly conserved hypusination domain in EheIF5A indicates an early establishment of the domain’s function and likely its PTMs throughout eukaryotic evolution.

### Recombinant EhDHS1 is active against eIF5A1/A2 while EhDHS2 is inactive

**Fig 3:**
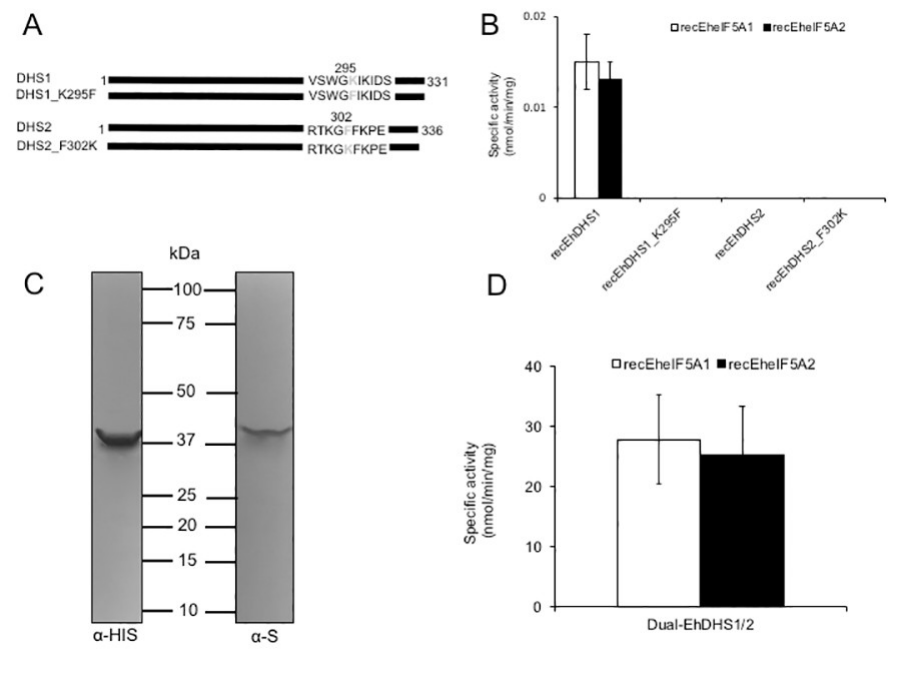
**Characterization of wild type, mutant and co-expressed forms of EhDHS1 and EhDHS2 (A)** Schematic representation of DHS1 and DHS2 mutant. Amino acid residues mutated are shown in grey. Amino acid positions are also shown. **(B)** Enzymatic activity of recombinant EhDHS1, EhDHS2, EhDHS1_K295F, and EhDHS2_F302K using EheIF5As substrates. The mean ± S.D. of three independent experiments performed in triplicate are shown. (**C**) Immunoblot analysis of co-expression of EhDHS1, which contains the amino-terminal His-tag, and EhDHS2, which contains the carboxyl-terminal S-tag, using anti-His and anti-S tag antibodies. **(D)** Specific activity of co-expressed recombinant EhDHS1 and EhDHS2 using EheIF5A1 and EheIF5A2 as substrates. The means ± standard deviations of three independent experiments performed in triplicate are shown.

To understand the role of the two EhDHS isotypes, enzymological characterization of recombinant EhDHS1 and 2 was performed. Recombinant EhDHS1, EhDHS2, EheIF5A1, and EheIF5A2 were produced at the level of 2.0–2.5% of the total soluble proteins in *E*. *coli* BL21 cells. SDS-PAGE analysis followed by Coomassie Brilliant Blue staining showed that the purified recombinant EhDHS1, EhDHS2, EheIF5A1, and EheIF5A2 proteins were present as apparently homogenous 37.2, 37.4, 18.6 and 17.1 kDa proteins, respectively, under reducing conditions (S3A Fig). The mobility of all recombinant proteins was consistent with the predicted size of the monomeric proteins with an extra 2.6 kDa histidine tag added at the amino terminus. We next tested the enzymatic activities of recombinant EhDHS1 and EhDHS2 using recombinant EheIF5A1 and EheIF5A2 as substrates. We found that EhDHS1 is active with the approximate specific activity of 0.015±0.003 nmol/min/mg with EheIF5A1 and 0.013±0.002 nmol/min/mg protein with EheIF5A2 (Fig 3B). The observed enzyme activity was 1000- to 2000-fold lower than the DHS activities reported from other organisms [19, 23], which may be explained in part by the fact that key residues involved in substrate binding are missing in EhDHS1. In contrast, recombinant EhDHS2 showed no detectable activity (Fig 3B), which is consistent with the fact that EhDHS2 lacks key amino acid residues including lysine, which is implicated in the formation of enzyme-substrate intermediates, as well as other amino acids involved in the spermidine binding (S1 Fig).

### The conserved lysine residue in the catalytic site is necessary, but not sufficient for DHS enzymatic activity

To better understand the physiological role of the apparently redundant, active and inactive EhDHS isotypes in *E*. *histolytica*, and, more specifically, to understand the reason for the lack of activity of EhDHS2, we made two EhDHS1 and EhDHS2 mutants, in which the lysine residue implicated for catalysis is replaced with phenylalanine in EhDHS1 or phenylalanine was replaced with lysine in EhDHS2, by site-directed mutagenesis (EhDHS1_K295F and EhDHS2_F295K) (Figs 3A and S3B). Both mutants were inactive (Fig 3B) with either EheIF5A1 or EheIF5A2 as a substrate, suggesting that the lysine residue in the enzyme-substrate intermediate site is essential but not sufficient for activity, and that substitutions of other amino acids responsible for spermidine and NAD^+^ binding also contribute to the lack of enzymatic activity in EhDHS2.

### EhDHS1 forms a complex with EhDHS2, which strongly enhances enzymatic activity of EhDHS1

We speculated if the enzymatically inactive EhDHS2 plays a regulatory role on the enzymatic activity harbored by EhDHS1, as previously reported in the DHS from *Trypanosoma brucei* [16]. We used a system that allows co-expression of two proteins in *E*. *coli* as described previously [24, 25]. Using pETDuet-1 vector, we were able to create a bacterial strain that expresses EhDHS1 with amino-terminal His-tag and EhDHS2 with carboxyl-terminal S-tag. Co-expression of EhDHS1 and EhDHS2 was verified by immunoblot analysis using anti-His and anti-S tag antibodies, respectively (Fig 3C). Recombinant His-tag DHS1 and S-tag DHS2 were copurified using Ni^2+^-NTA resins as described in the Materials and methods section. The purified proteins were present in approximately equimolar amounts, indicating that EhDHS1 and EhDHS2 form a stable complex with a ratio of one to one. DHS activity of the dimeric EhDHS1/EhDHS2 proteins was measured using EheIF5A1 or EheIF5A2 as substrates. We detected an approximately 2,000-fold increase in the enzymatic activity when EhDHS1 and EhDHS2 were co-expressed, as compared to EhDHS1 alone (27.8±7.4 nmol/min/mg protein with EheIF5A1 and 25.2±8.3 nmol/min/mg protein with EheIF5A2) (Fig 3D).

### EhDHS1/2 forms a ~158 kDa protein complex

**Fig 4:**
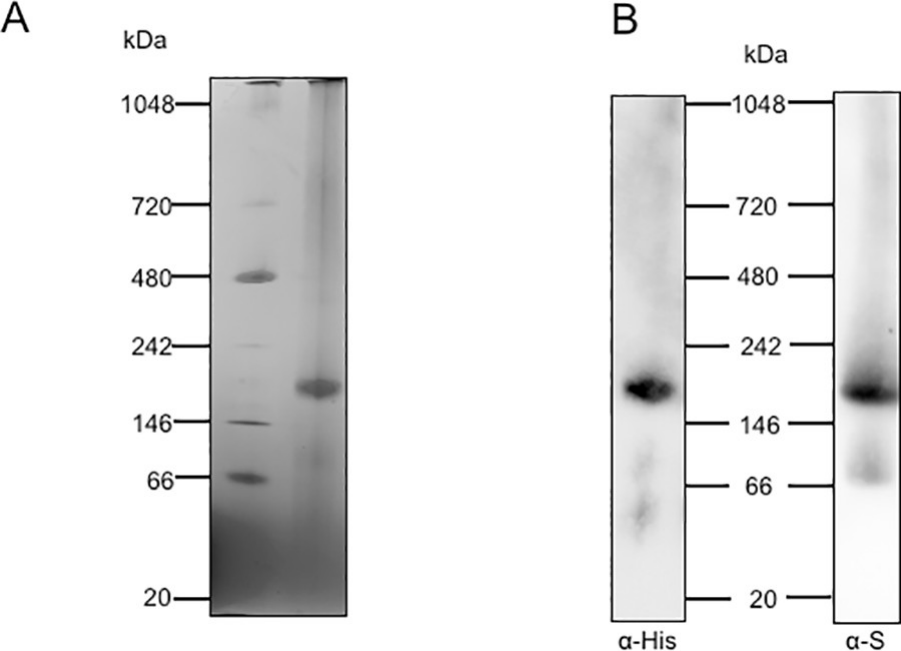
Determination of the size of the complex of EhDHS1 and EhDHS2. **(A)** Purified Recombinant EhDHS1 and 2 were co-expressed, copurified, and were subjected to 4–16% BN-PAGE. After electrophoresis the gel was destained. Native page marker (Invitrogen) was used as a protein standard. **(B)** Immunoblot analysis following BN-PAGE of co-expressed N-terminal His-tagged EhDHS1 and C-terminal S-tagged EhDHS2, using anti-His and anti-S tag antibodies respectively.

To determine the size of the EhDHS1/2 complex, we performed blue native polyacrylamide gel electrophoresis (BN-PAGE) of EhDHS1/2 that had been purified together after co-expression in bacteria (Fig 4A), followed by immunoblot analysis using anti-His and S-tag antibodies, respectively (Fig 4B). We detected a band suggestive of a complex containing both EhDHS1 and 2 (Fig 4A). The size of the complex is approximately 158 kDa, suggesting that EhDHS1 and 2 formed a tetrameric complex.

### Both *EhDHS1* and *EhDHS2* genes appear to be essential for *E*. *histolytica* growth

**Fig 5:**
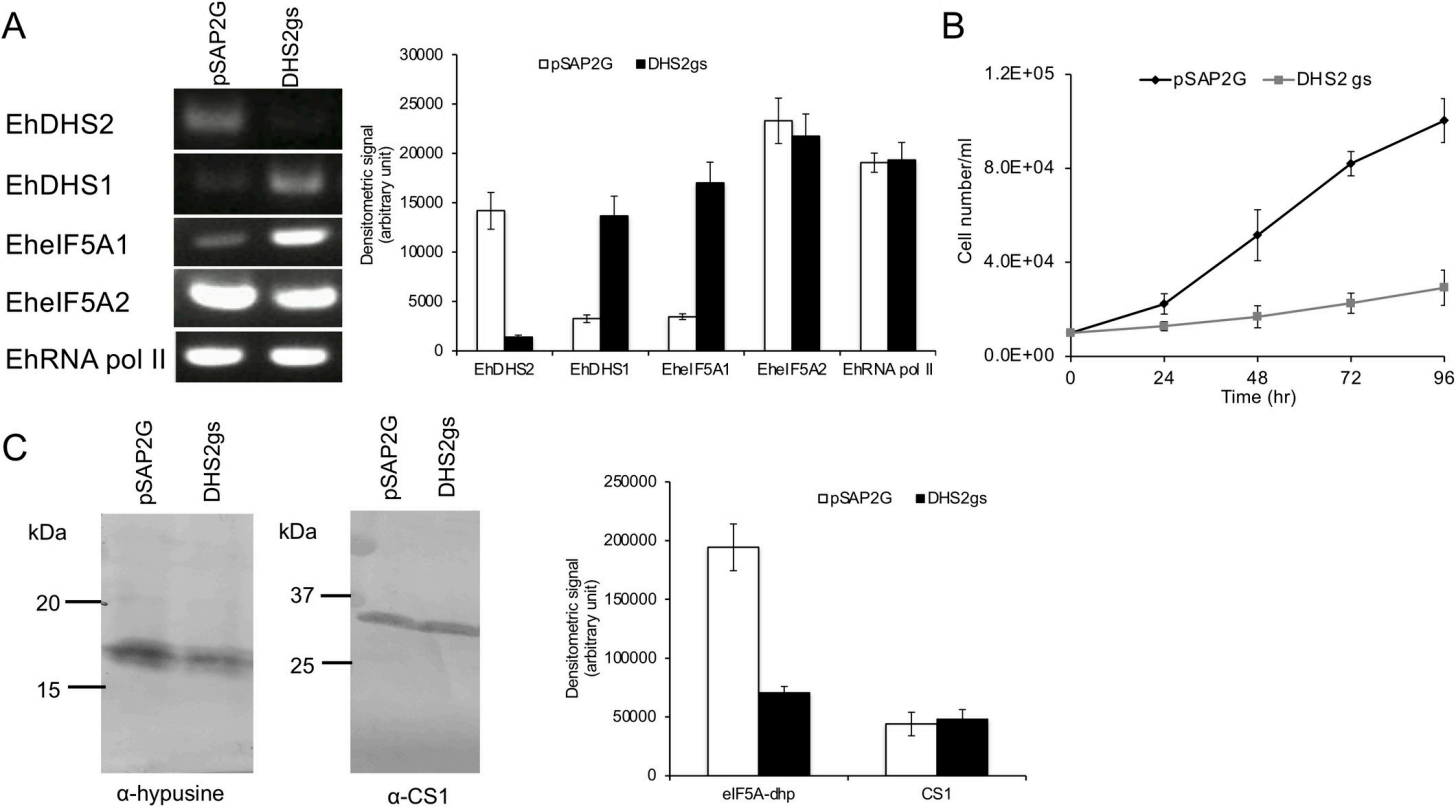
Effects of *EhDHS2* gene silencing on gene expression of related genes and growth of *E*. *histolytica* trophozoites. **(A)** Evaluation of gene expression by semi-quantitative RT-PCR analysis of *EhDHS2* gene silenced transformant. The steady-state levels of transcripts of *EhDHS1*, *EhDHS2*, *EheIF5A1*, *EheI5FA2* and *EhRNA pol II* genes were measured in trophozoites of G3 strain transfected with either empty vector (psAP2G) or the *EhDHS2* gene silencing plasmid (psAP2G-DHS2). cDNA from the generated cell lines (psAP2G and DHS2gs strains) was subjected to 30 cycles of PCR using specific primers for the *DHS2*, *DHS1*, *eIF5A1*, *eI5FA2* and *RNA pol II* genes. RNA polymerase II served as a control. PCR analysis of samples without reverse transcription was used to exclude the possibility of genomic DNA contamination. The densitometric quantification of the bands, shown in the right graph, was performed by Image J software, and the expression level of *EhDHS1*, *EhDHS2*, *EheIF5A1*, *EheIF5A2*, and *EhRNA pol II* was expressed in arbitrary units. **(B)** Growth kinetics of control (pSAP2G) and *EhDHS2* gene silenced (DHS2gs) transformants. Approximately 60,000 amoebae in the logarithmic growth phase were inoculated into 6 mL fresh culture medium and amoebae were then counted every 24 hr. Data shown are the means ± standard deviations of five biological replicates. **(C)** Immunoblot analysis of control (pSAP2G) and EhDHS2 gene silenced (DHS2gs) transformants using hypusine antibody. Total cell lysate was electrophoresed on a 15% SDS-PAGE gel and subjected to an immunoblot assay with hypusine antibody and CS1 (loading control) antiserum. The intensity of the bands corresponding to hypusinated EheIF5A and CS1 was measured by densitometric quantification, analyzed by Image J software, and is shown in arbitrary units in the right graph.

To investigate the role of DHS1 and DHS2 in *E*. *histolytica*, we exploited antisense small RNA-mediated epigenetic gene silencing to repress the *EhDHS1* and *EhDHS2* genes in *E*. *histolytica* G3 strain [26, 27]. However, our repeated attempts to create a transformant in which *EhDHS1* gene expression was repressed by gene silencing failed, suggesting the essentiality of the gene. In contrast, we successfully gene silenced *EhDHS2* (Fig 5A) and found that *EhDHS2* gene-silenced transformants displayed severe growth defect (Fig 5B). Interestingly, we found that in *EhDHS2* gene-silenced strain, the steady-state levels of transcripts of *EhDHS1* and *EheIF5A1* were up regulated approximately 4-fold (Fig 5A). We next checked whether *DHS2* gene silencing affects the hypusination levels in the cells. Using anti-hypusine polyclonal antibody [28] we examined the hypusination levels in *EhDHS2* gene-silenced and control transformants. We found that hypusination levels decreased by 64% (Fig 5C), suggesting that EhDHS2 plays, likely via interaction with and activation of EhDHS1, an important role in maintaining the hypusination levels in the cell.

### Intracellular distribution of EhDHS1, EhDHS2, EheIF5A1, and EheIF5A2 and hypusine modification of EheIF5A proteins

**Fig 6:**
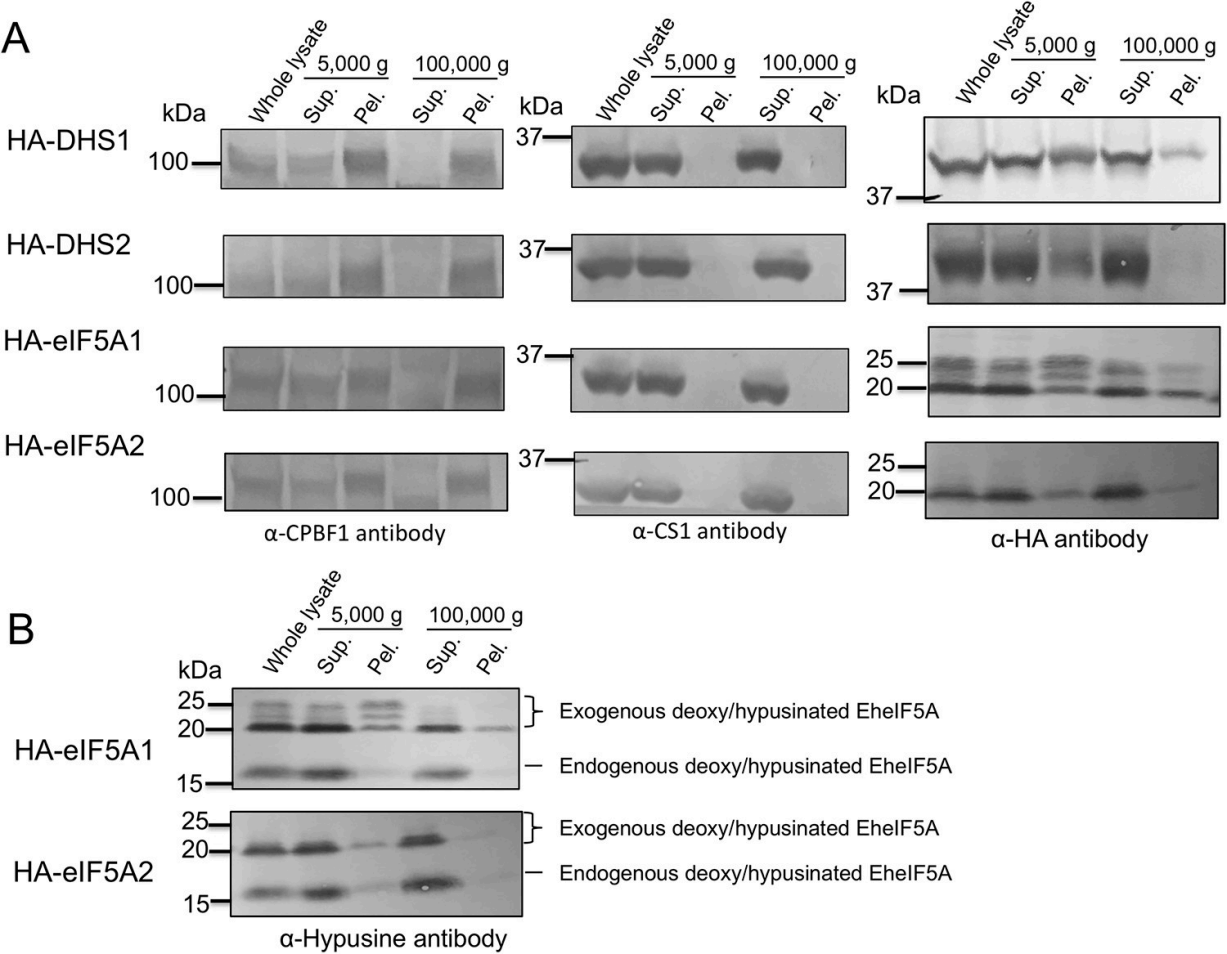
Cellular distribution of EhDHS and EheIF5A isoforms and post translational modification of EheIF5A1/2. **(A)** Cellular fractionation of EhDHS1, EhDHS2, EheIF5A1, and EheIF5A2. Trophozoites of the transformants expressing HA-tagged EhDHS1, EhDHS2, EheIF5A1, and EheIF5A2 were fractionated as described in Materials and Methods, and subjected to immunoblot analysis using anti-HA monoclonal antibody, anti-hypusine, anti-CPBF1, and anti-CS1 polyclonal antisera. CPBF1 and CS1 served as control of organelle and cytosolic proteins, respectively. **(B)** PTMs of HA tagged EheIF5A. Approximately 30 μg of total lysates from the transformants expressing HA-tagged EheIF5A1 and EheIF5A2 were subjected to SDS-PAGE under reducing conditions and immunoblot analysis using anti-hypusine antibody.

To examine intracellular distribution of EhDHS1, EhDHS2, EheIF5A1, and EheIF5A2 in trophozoites, we established transformant lines expressing EhDHS1 and EhDHS2 with hemagglutinin (HA) tag at the amino terminus (HA-EhDHS1, HA-EhDHS2, HA-EheIF5A1, and HA-EheIF5A2). The expression of these proteins in trophozoites was confirmed by immunoblot analysis with anti-HA antibody (S4 Fig). We first examined the distribution of EhDHS1, EhDHS2, EheIF5A1, and EheIF5A2 in trophozoites by immunoblotting using lysates produced by a Dounce glass homogenizer, and centrifugation at 5,000 g and subsequently at 100,000 g. We found that EhDHS1, EhDHS2, EheIF5A1, and EheIF5A2 were mainly contained in the soluble fraction of centrifugation at 100,000 g, together with the cytosolic soluble protein CS1 [29] (Fig 6A). We also found that EhDHS1 and EheIF5A1 (also in a lesser amount, EhDHS2 and EheIF5A2) were associated with the pellet fraction after centrifugation at 100,000 g, together with the vesicular membrane protein CPBF1 [30] (Fig 6A). These data indicate that EhDHS1, EhDHS2, EheIF5A1, and EheIF5A2 were mainly present in the cytosol, but they are also partially localized to some organelle(s)/compartment(s). Note that HA-EheIF5A1 is present as multiple bands, suggesting PTMs and those multiple forms are present in varying proportions among different fractions.

We further examined potential PTM of EheIF5A1 and EheIF5A2 using HA-EheIF5A1 and HA-EheIF5A2 overexpressing strains. Using anti-hypusine antibody, we detected four bands in HA-EheIF5A1-expressing trophozoites; the top three 20–25 kDa bands correspond to the exogenously expressed HA-EheIF5A1 while the bottom 17 kDa band corresponds to the endogenous EheIF5A (Fig 6B). In HA-EheIF5A2-expressing transformants, we detected two bands corresponding to exogenous and endogenous EheIF5A respectively (Fig 6B). However, we detected only a single band corresponding to the endogenous EheIF5A using anti-hypusine antibody (Fig 6B). These data indicate that both EheIF5A1 and EheIF5A2 undergoes hypusination, but only EheIF5A1 is additionally subjected to PTMs other than hypusination. It was previously reported in other organisms that besides hypusination, eIF5As are post-translationally modified by acetylation [21], sulfation [22], phosphorylation [31, 32] and glycosylation [32]. However, the nature of modifications in HA-EheIF5A1 expressing cells remains unknown. Our attempt to characterize PTMs on eIF5A1 and eIF5A2 that had been immunoprecipitated with anti-HA antibody followed by trypsinization and mass spectrometric analysis, failed despite repeated trials. The coverage of detected peptides from the immunoprecipitated samples on eIF5A1/2 ranged 75–85%. Potential sites of hypusination [(D)(T)GKHGHAK] in eIF5A1 and eIF5A2 were not detected as either unmodified or modified by manual inspection of mass spectrometric data (Fig 2B). Peptides containing either Lys62 in EheIF5A1 or Lys53 in EheIF5A2 were not detected by mass spectrometry.

We found that both EheIF5A isotypes in lysates from amebic trophozoites were recognized by commercial anti-hypusine antibody via immunoblot analysis (S4 Fig), suggesting that eIF5A proteins in trophozoites may be hypusinated. However, DOHH which catalyzes the final step of the formation of mature hypusinated eIF5A in other organisms (Fig 1), seems to be missing in the *E*. *histolytica* genome (https://amoebadb.org/amoeba/). To verify if anti-hypusine polyclonal antibody [28] used in this study recognizes hypusinated and/or deoxyhypusinated forms of eIF5A, we performed immunoblot analysis of recombinant eIF5A1 and eIF5A2 incubated in vitro with a combination of recombinant EhDHS1 and EhDHS2 (S5 Fig). We found that the deoxyhypusinated form of eIF5A1 (lane 4) and eIF5A2 (lane 5) were detected by anti-hypusine antibody only when they had been co-incubated with the recombinant DHSs and spermidine (S5 Fig), suggesting that the anti-hypusine antibody can also recognize deoxyhypusinated eIF5A. Thus, the nature of protein modifications, that is, whether eIF5A is only deoxyhypusinated or fully hypusinated in amebic trophozoites, still remains elusive.

### Only eIF5A2 protein is expressed in trophozoites

In order to examine the levels of expression of each isoform of eIF5As in *E*. *histolytica* trophozoites, polyclonal antisera were raised against purified recombinant EheIF5A1 and EheIF5A2. The cell lysates of *E*. *histolytica* transformant cell lines expressing EheIF5A1 and EheIF5A2 with the HA tag fused at the amino terminus (HA-EheIF5A1 and HA-EheIF5A2) were analyzed by immunoblotting with antisera raised against EheIF5A1 and EheIF5A2. We confirmed specificities of these antisera using corresponding cell lines expressing EheIF5A1 and EheIF5A2 proteins (S6 Fig). To our surprise, only EheIF5A2, but not EheIF5A1, was detected in wild type trophozoites as well as all the transformants (S6 Fig). Our previous transcriptome analysis using DNA microarray [33] also suggests that *EheIF5A2* gene is more abundantly transcribed than *EheIF5A1* gene.

### *EheIF5A2* gene silencing causes up regulation of *EheIF5A1* gene expression and also inhibits protein translation

**Fig 7:**
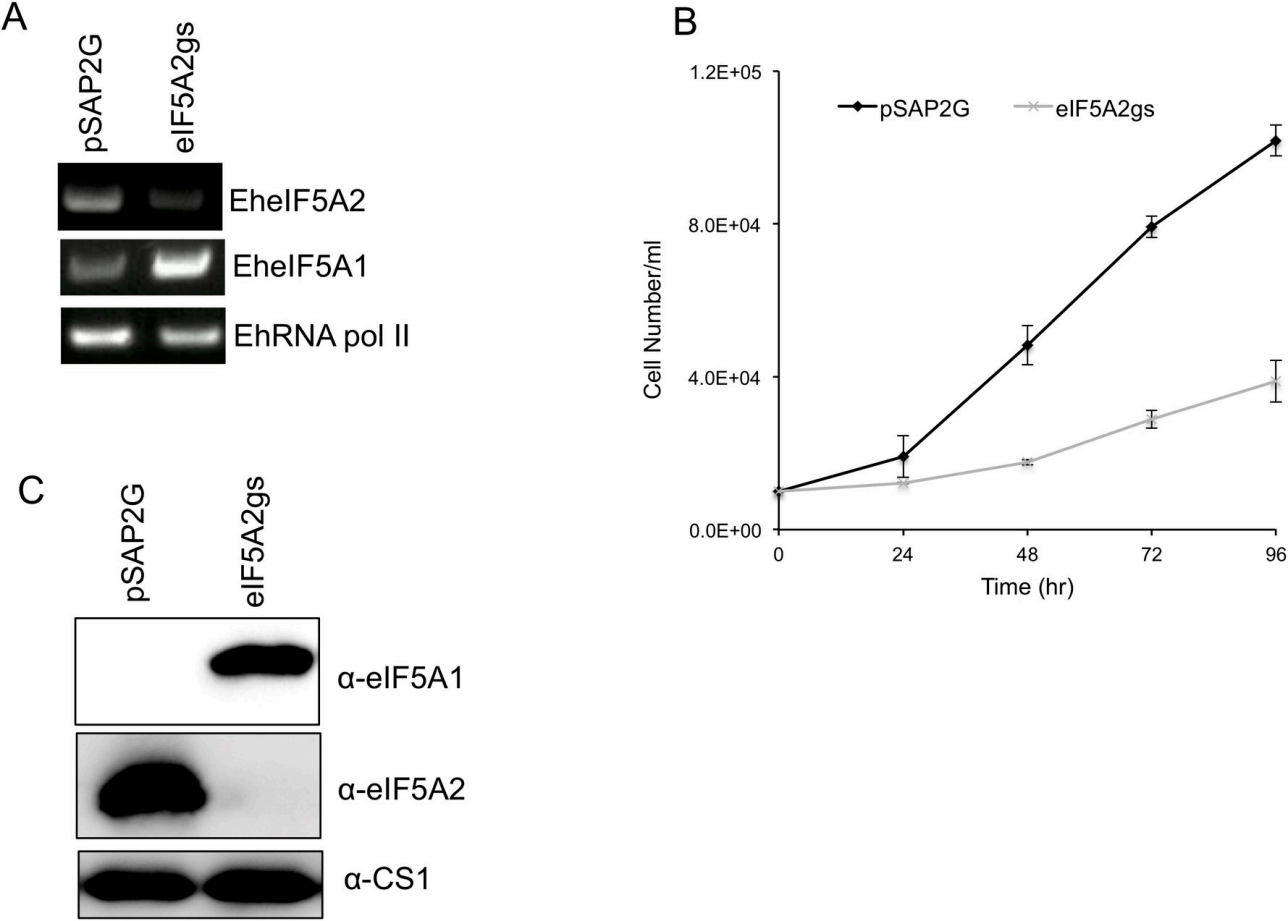
Silencing of *EheIF5A2* caused growth retardation and up regulation of EheIF5A expression. **(A)** Evaluation of gene expression by semi-quantitative RT-PCR analysis of *EheIF5A2* gene silenced transformant. The steady-state levels of transcripts of *EheIF5A1*, *EheIF5A2*, and *EhRNA pol II* genes were measured in trophozoites of G3 strain transfected with either empty vector (psAP2G) or the *EheIF5A2* gene silencing plasmid (psAP2G-eIF5A2). cDNA from the generated cell lines (psAP2G and *EheIF5A2*gs strains) was subjected to 25 cycles of PCR using specific primers for the *eIF5A1*, *eI5FA2* and *RNA pol II* genes. RNA polymerase II served as a control. PCR analysis of samples without reverse transcription was used to exclude the possibility of genomic DNA contamination. **(B)** Growth kinetics of *EheIF5A2* gene-silenced (*EheIF5A2*gs) and control (pSAP2G) transformants. Approximately 60,000 amoebae in the logarithmic growth phase were inoculated into 6 ml fresh culture medium and amoebae were then counted every 24 hr. Data shown are the means ± standard deviations of five biological replicates. **(C)** An immunoblot analysis of *EheIF5A2*gs and control (pSAP2G) transformants. Total cell lysates were electrophoresed on a 15% SDS-PAGE gel and subjected to an immunoblot assay with antibodies raised against EheIF5A1 and EheIF5A2.

In order to better understand the role of EheIF5A2, we utilized gene silencing to repress the *EheIF5A2* gene in *E*. *histolytica* G3 strain. *EheIF5A2* transcript was undetectable in EheIF5A2gs line (Fig 7A). Interestingly, we found that the steady-state level of *EheIF5A1* transcript increased upon *EheIF5A2* gene silencing (Fig 7A). We further examined eIF5A1 and eIF5A2 expression at the protein level in EheIF5A2gs and control strains. Immunoblot analysis using anti-EheIF5A1 and anti-EheIF5A2 antibodies showed that eIF5A2 protein was undetected by anti-EheIF5A2 antibody while eIF5A1 protein was detected in the EheIF5A2gs strain, suggesting compensatory expression of EheIF5As for survival (Fig 7C). However, the compensatory upregulation of *eIF5A1* gene expression may not be sufficient to completely rescue the function of eIF5A2 because EheIF5A2gs strain still displayed growth defect (Fig 7B).

**Fig 8:**
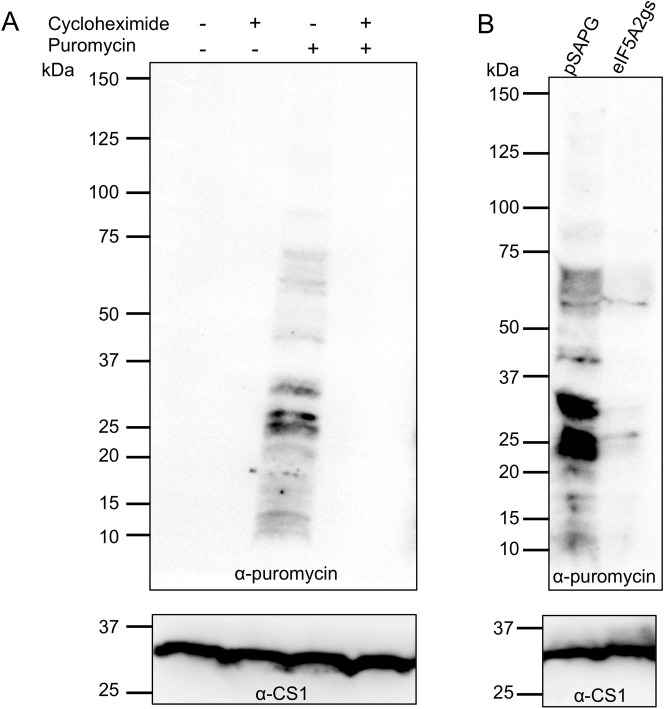
SUnSET analysis of active protein synthesis. **(A)** SUnSET analysis of wild type strain. Trophozoites of *E*. *histolytica* clonal strain HM-1:IMSS cl 6 were incubated in normal growth medium with 100 μg/ml cycloheximide, 10 μg/ml puromycin, or both. Cell lysates were subjected to SDS-PAGE and immunoblot using antibody specific for puromycin. Note that trophozoites incorporated puromycin into proteins (P). The incorporation was blocked by cycloheximide treatment (C + P). These data validated the SUnSET analysis for assessing protein synthesis in *E*. *histolytica*. **(B)** SUnSET analysis of *EheIF5A2*gs strain. Trophozoites from *EheIF5A2*gs and control (pSAP2G) strains were subjected to SUnSET analysis as in (A). Equal protein loads were confirmed with CS1 antibody (lower panels). Representative data for at least 3 separate trials are shown.

Since *EheIF5A2* gene silencing caused growth defect, we next examined whether it also affects protein synthesis per se. We utilized SUrface SEnsing of Translation (SUnSET) technique to monitor protein synthesis in *E*. *histolytica* [34]. To first validate that SUnSET is applicable to *E*. *histolytica*, amebic trophozoites were incubated with puromycin before or after incubation with cycloheximide, an inhibitor of de novo protein synthesis. Immunoblotting using anti-puromycin antibody revealed that puromycin was readily incorporated into proteins in *E*. *histolytica* (Fig 8A). The incorporation was dependent on de novo protein synthesis since no background staining was detected in the lysates from cycloheximide-treated cells (Fig 8A). We compared the protein synthesis efficiency in EheIF5A2gs and control strains. We found that in EheIF5A2gs strain, puromycin incorporation was almost completely abolished as compared to control (Fig 8B), indicating ceased protein synthesis caused by EheIF5A2 gene silencing.

### eIF5A1 may play an important role in stage conversion

It was previously shown that eIF5A plays an important role in mouse embryogenesis and cell differentiation [35]. *Entamoeba* also undergoes differentiation between the proliferative trophozoite and the dormant cyst (encystation and excystation). We examined the transcriptomic profiles of *E*. *invadens*, a parasite of reptiles, which mimics the biology and pathophysiology of *E*. *histolytica*, and easily undergoes encystation and excystation in vitro [36]. We identified two DHS homologs, which show 34% mutual identity, in the *E*. *invadens* genome database (https://amoebadb.org/amoeba/). Similar to *E*. *histolytica*, one *E*. *invadens* DHS (EiDHS1) ortholog contains the key catalytic lysine residue, while the residue is not conserved in the other isotype (EiDHS2) (S1 Fig). EiDHS1 shows 71% amino acid identity to EhDHS1 and EiDHS2 exhibits 76% amino acid identity to EhDHS2. We further identified three eIF5A orthologs (EIN_344230, EIN_129200, and EIN_296010). We named them as EieIF5A1 (EIN_344230), EieIF5A2 (EIN_129200), and EieIF5A3 (EIN_296010) in descending order of percentage amino acid identity with *E*. *histolytica* eIF5A1. EieIF5A1 showed 50% amino acid identity to EheIF5A1 and EieIF5A2 exhibit 73% amino acid identity to EheIF5A2, while EieIF5A3 shows only 23% and 15% amino acid identity to *E*. *histolytica* eIF5A1 and eIF5A2, respectively (S7A Fig). A multiple sequence alignment of three EieIF5A isotypes with two EheIF5A and two human eIF5A isoforms, by the ClustalW program (http://clustalw.ddbj.nig.ac.jp/top-e.html), shows that the two glycine residues adjacent to lysine (red) are also conserved in EieIF5A (S7B Fig). As mentioned above, this Gly-X-Y-Gly motif is reported to be critical for β turn structure and the proper orientation of the deoxyhypusine/hypusine side chain [20]. Lysine residues at amino acid positions 52 (EieIF5A1), 50 (EieIF5A2), and 66 (EieIF5A3) predicted for hypusination are conserved in all three EieIF5A (S7B Fig). Phylogenetic analysis revealed that eIF5A1 and eIF5A2 homologs from *Entamoeba* species form independent and well clustered clades, while EieIF5A3 represents a divergent member as it is well separated from all eIF5As homologs from representative organisms (S8 Fig).

In order to investigate whether *E*. *invadens* eIF5As is (deoxy)hypusinated, we used anti-EheIF5A1, EheIF5A2, and hypusine antibodies to examine expression and modification of EieIF5A isoforms. We detected a single band corresponding to EieIF5A2, but not EieIF5A1, using anti-EheIF5A antibodies (S9 Fig). EieIF5A1 did not appear to be expressed in *E*. *invadens* trophozoites, similar to *E*. *histolytica*. However, one should be cautious in the interpretation of the data because heterologous antibodies were used (note that the mutual amino acid identity between EieIF5A2 and EheIF5A2 is 73%, whereas it is only 50% between EieIF5A1 and EheIF5A1). These data indicate that at least EieIF5A2 was hypusinated or deoxyhypusinated. It is noticeable that the band intensity of EieIF5A2 detected with anti-EheIF5A2 was lower than that detected with anti-hypusine antibody, suggesting that a significant proportion, if not all, of EieIF5A2 is hypusinated or deoxyhypusinated.

To determine whether eIF5As are involved in the differentiation process of *E*. *invadens*, we carried out in vitro encystation of *E*. *invadens* using the methods described previously [37]. Approximately 90% of the trophozoites differentiated into sarkosyl-resistant cysts within 120 hr (S10A Fig). Under the conditions, EieIF5A2 was abundantly expressed in *E*. *invadens* trophozoites similar to *E*. *histolytica*, while neither *EieIF5A1* nor *EieIF5A3* expression was detected in trophozoites (S10B Fig). In the course of encystation, the expression levels of *EieIF5A1-3* remained unchanged (S10B Fig), suggesting these genes are not involved in encystation of *Entamoeba*.

**Fig 9:**
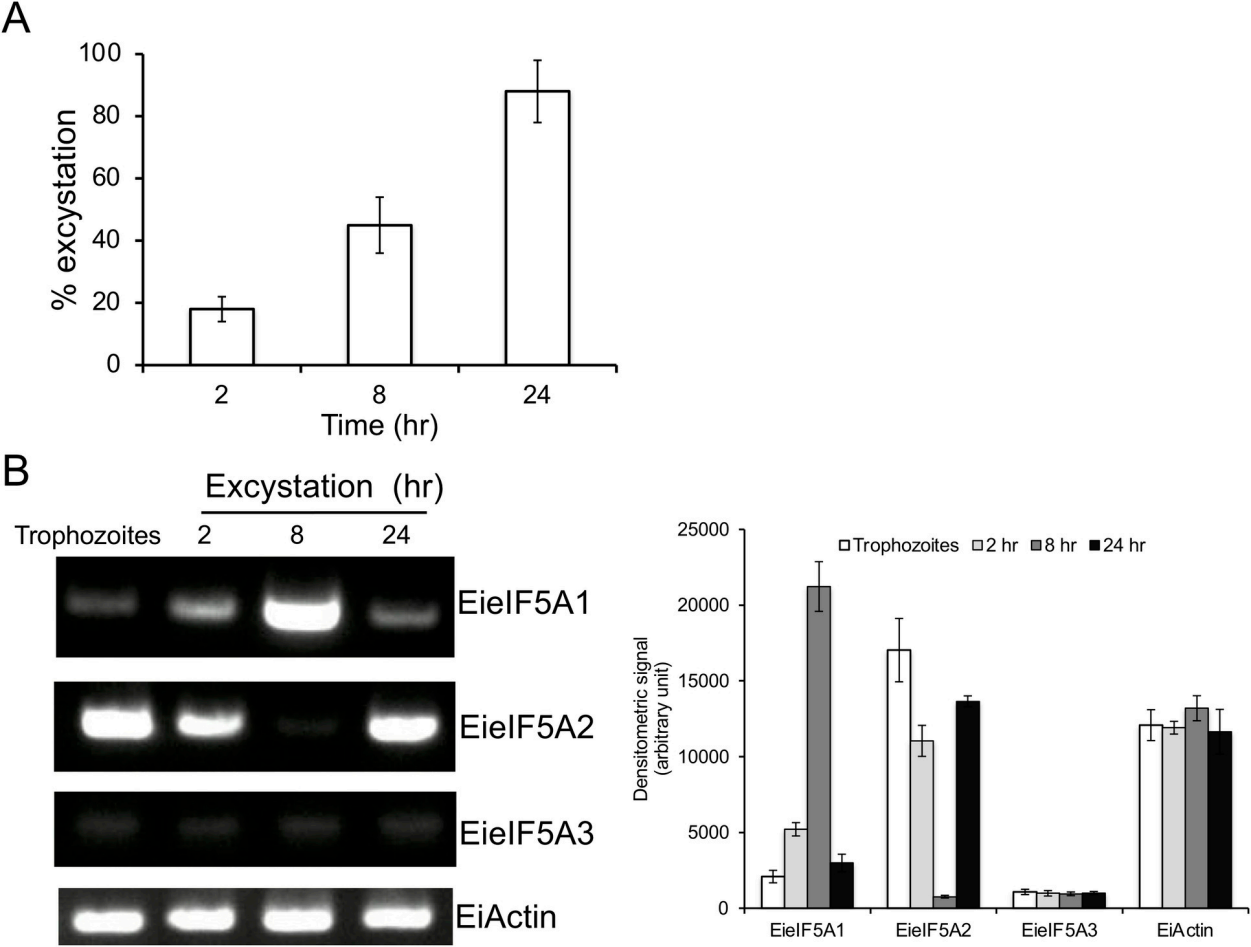
Steady state transcript levels of EieIF5A isoforms during excystation. **(A)** Kinetics of excystation. Cysts were incubated at the indicated times, cysts and trophozoites were harvested and the excystation percentage was determined. The excystation efficiency was determined by counting trophozoites and cysts in a haemocytometer and experiments were performed in duplicate. Data shown are the means ± standard deviations of two independent experiments. **(B)** Semi-quantitative RT-PCR analysis of *EieIF5A1*, *EieI5FA2*, *EieIF5A1* and *EiActin* genes during excystation. cDNA from 4 time points during excystation was subjected to 30 cycles of PCR using specific primers for *eIF5A1*, *eI5FA2*, *eIF5A3*, and *actin* genes. *Actin* gene served as a control. PCR from samples without reverse transcription served as controls to exclude the possibility of genomic DNA contamination. The densitometric quantification of the bands, shown in the right graph, was performed by Image J software, and the expression level of *EieIF5A1*, *EieIF5A2*, and *EieIF5A3 and EiActin* was expressed in arbitrary units.

Next, we investigated whether eIF5As are involved in excystation. We found that approximately 45% and 88% of the cysts transformed into trophozoites at 8 and 24 hr, respectively (Fig 9A). In the course of excystation, the steady-state level of *EieIF5A1* transcript was increased and peaked at 8 hr, while that of *eIF5A2* transcript was transiently abolished at 8 hr, but increased to the level similar to that in the cyst (Fig 9B), suggesting that eIF5A1 plays an excystation-specific role in *Entamoeba*. The transcript level of *EieIF5A3* gene, which is not expressed in trophozoites, remained unchanged throughout the excystation process.

## Discussion

### eIF5A plays an important role in proliferation and potentially in differentiation of *E*. *histolytica*

In this study, we have demonstrated that eIF5A and its PTMs are essential for both proliferation and differentiation in *Entamoeba*. Stage conversion is a key biological process needed for transmission of eukaryotic pathogens [36–38]. We have shown that *Entamoeba* eIF5A is involved in excystation, conversion from the cyst to the trophozoite, but not in encystation. *E*. *invadens*, used as an in vitro differentiation model, possesses three isoforms of eIF5A (S7 Fig). Of the two *E*. *invadens* isoforms orthologous to *E*. *histolytica* isoforms (EieIF5A1 and EieIF5A2), *EieIF5A1* gene was strongly upregulated only at 8 hr post excystation induction, strictly in a time-dependent fashion, when *EieIF5A2* expression was conversely repressed (Fig 9B). In contrast, *EieIF5A2* gene was constitutively expressed in trophozoites, encysting trophozoites, and cysts. These data intriguingly indicate a specific role of EieIF5A1 in this very narrow time window of excystation. It needs to be elucidated in the future how eIF5A1 and eIF5A2 are coordinately involved in excystation: whether eIF5A1 is involved in protein synthesis per se or other cellular functions, and in what cellular compartments. In humans, two eIF5A isoforms are known to exert specialized activities in certain tissues and cancer cells in addition to basic overlapping functions [39]. It was suggested that the divergent C-terminal domain of eIF5A isotypes may be involved in an interaction with alternate binding partners and is responsible for homo-oligomerization as noted for human eIF5A1 [40] and its archaeal counterpart [41].

### Catalytic and regulatory roles of two EhDHS isoforms

eIF5A is the only protein which undergoes hypusination in nature. No pathway is known for the synthesis of this modified amino acid. We have identified proteins responsible for deoxyhypusine modification of eIF5A but failed to identify proteins acting as the final enzyme to complete eIF5A hypusination. Whether DOHH is present in *E*. *histolytica* and, if so, its identity remains elusive. We have shown that two isoforms of EhDHS play catalytic and regulatory roles in eIF5A modifications. Most eukaryotes possess either only a single DHS or two closely related active DHSs. There are a few exceptions known other than *Entamoeba*: *Trypanosoma brucei* [16] and *Leishmania* [17], both of which, together with *E*. *histolytica*, possess two DHS, one catalytically active and one inactive [16]. In *T*. *brucei*, it was reported that two each of the catalytically active and inactive isotypes formed a heterotetrameric complex, and DHS activity increased 3000-fold by heterotetramer formation [16]. It was also shown in *T*. *brucei* that the loss of one of the isotypes led to instability of the complex, suggesting both DHS isotypes were necessary. Furthermore, both *EhDHS1* and *2* genes were essential for parasite growth [16]. In contrast to what was reported in *T*. *brucei*, we found that gene silencing of *EhDHS2* (inactive isoform) did not cause loss of either EhDHS1 protein or hypusinated eIF5A (Fig 5C). It is conceivable that in *EhDHS2* gene-silenced strain, the decreased activity of EhDHS1 or instability of the EhDHS1/2 complex is partially compensated by transcriptional upregulation of *EhDHS1* and *eIF5A1*genes (Fig 5A).

### Possible posttranslational modifications of *E*. *histolytica* eIF5A other than hypusination

Hypusination was shown to play a critical role in the regulation of eIF5A [6]. In addition to hypusination, other modifications, such as phosphorylation [31, 32], glycosylation [32], acetylation [21, 42], transglutaminylation [43], and sulfation [22] of eIF5A have been described, but very little is known about their roles. In *E*. *histolytica*, both eIF5A1 and eIF5A2 are deoxyhypusinated/hypusinated, and only eIF5A1 undergoes other PTMs. The reason of isotype-specific PTMs other than hypusination on eIF1A1, but not on eIF1A2, remains unclear and needs future investigation. However, as shown in Fig 2B, EheIF5A1 contains residues, which are post-translationally modified by acetylation (K80) and sulfation (Y81) in human eIF5A [21, 22], neither of which is conserved in EheIF5A2. However, the nature of protein modification in HA-EheIF5A1-expressing cells in amebic trophozoites, still remains elusive. It has been recently reported that human eIF5A rapidly translocates to the Golgi apparatus and is further modified by tyrosine-sulfation in the *trans*-Golgi prior to secretion [22]. Secreted tyrosine sulfated-eIF5A mediates oxidative stress-induced apoptosis by acting as a pro-apoptotic ligand [22]. It has also been reported in *T*. *vaginalis* that eIF5A is phosphorylated at serine 2 in a stretch of the consensus sequence present at the amino terminus (MSSAEEEV) [44]. A similar sequence (MSSNGSDN) is also present at the amino terminus of EheIF5A2, but not in EheIF5A1, suggesting that EheIF5A2 may be phosphorylated (Fig 2B). It has been reported that in maize, a serine 2 at the amino terminus (MSDSEEHH) of eIF5A is phosphorylated by the catalytic α-subunit of the casein kinase 2 (CK2) [31]. This phosphorylation is also known to play a role in the regulation of the nucleo-cytoplasmic shuttling of eIF-5A in plant cells [45]. However, we failed to detect phosphorylation of EheIF5A2 using anti-phospho serine antibody by immunoblot analysis.

### Localization of eIF5A may be attributable to posttranslational modifications

We found by cellular fractionation that EhDHS1, EhDHS2, EheIF5A1, and EheIF5A2 are associated with the 5,000 g pellet and 100,000 g pellet fractions, which contain the nucleus, the plasma membrane, large vacuoles (the former fraction), mitosomes, the endoplamsic reticulum, and lysosomes (the latter), although these four proteins were mostly associated with the soluble cytosolic fraction (Fig 6A). Intriguingly, three hypusinated bands corresponding to EheIF5A1 were mainly detected in the 5,000 g membrane fraction using anti-hypusine antibody, and the band patterns of hypusinated EheIF5A1 differed in 5,000 g pellet and other fractions (note the middle band is only detected in 5,000 g pellet), suggesting that the those heterogeneously modified eIF5A1 proteins are associated with the nucleus, the plasma membrane, and/or heavy membranes. Such PTMs on EheIF5A1 seem to modulate its functions by targeting EheIF5A1 to different cellular compartments to impair the interaction with its partners as demonstrated for human eIF5A [46]. The subcellular localization of eIF5A and its nuclear-cytoplasmic shuttling in other organisms remain controversial [47]. It has been reported that eIF5A is localized to the ER in multiple mammalian cell lines [48], while other studies have shown that eIF5A shuttles between the nucleus and the cytoplasm, and occasionally co-localizes with the nuclear pore complex [49]. It was also demonstrated that eIF5A enters the nucleus through passive diffusion and is exported from the nucleus to the cytoplasm in a CRM1(chromosomal maintenance 1, also known as exportin1)-dependent manner [50]. It was also shown that exportin 4, an importin β family receptor, is responsible for the nuclear export of eIF5A [51, 52]. Genome survey of *E*. *histolytica* suggest that exportin 1 (EHI_107080) and exportin T (EHI_029040) are encoded in the genome. It has been recently reported that exogenous eIF5A lacking hypusination tends to localize to the nucleus, compared to fully hypusinated endogenous eIF5A that mainly localizes to the cytoplasm, but can be translocated to the cytoplasm when fully hypusinated [53], suggesting that hypusination promotes a nucleus-to-cytoplasm transport. To evaluate the localization of HA*-*tagged eIF5A1 and eIF5A2 proteins, we performed immunofluorescence analysis using anti-HA and anti-CS1 (cytosolic control) antibodies. We found that EheIF5A1 and EheIF5A2 were present in the cytoplasmic fractions of *E*.* histolytica* cells (S11A Fig). In order to better understand the distribution of endogenous eIF5A1 and eIF5A2 levels, we have used control (only eIF5A2 expressed) and eIF5A2gs strain (only eIF5A1 expressed) (Fig 7C) and then performed immunofluorescence analysis using EheIF5A1 and EheIF5A2 antibodies, and also Hoechst for nuclear staining. Nevertheless, our immunofluorescence using imaging failed to detect nuclear localized eIF5A and suggests that both proteins are present in the cytoplasm of *E*. *histolytica* (S11B Fig).

### Multiplicity and essentiality of eIF5A

We showed that eIF5A2, but not eIF5A1, is constitutively expressed in trophozoites, although both isoforms harbor the hypusine modification **(**Fig 7C). Two or more *eIF5A* genes are present in various eukaryotes including fungi, plants, vertebrates, and mammals [54, 55]. In fish, amphibians, and birds, the two *eIF5A* genes seem to be co-expressed [56]. Humans have two eIF5As isoform however their biological functions are significantly different. One eIF5A isoform in humans is abundantly expressed in most cells, essential for cell proliferation, while the other isoform is undetectable or only weakly expressed in most cells and tissues, but highly expressed in various cancerous cells, and thus suggested to be associated with cancers [57, 58].

Gene disruption and mutation studies in yeast and higher eukaryotes indicate the essentiality of eIF5A and its deoxyhypusine/hypusine modifications [59]. In *Saccharomyces cerevisiae*, simultaneous disruption of two *eIF5A* genes or inactivation of the single *DHS* gene led to growth arrest, and substitution of one of two eIF5As with eIF5A K50R mutant also caused growth arrest [60, 61]. Interestingly, *EheIF5A2* gene silencing resulted in the compensatory up regulation of *EheIF5A1* gene expression (Fig 7A and 7C), suggesting that the role of eIF5A1 and eIF5A2 is interchangeable to some extent. Despite the observed *eIF5A1* upregulation, *EheIF5A2* gene-silenced strain showed defect in protein synthesis, suggesting EheIF5A1 has very limited role in protein synthesis and EheIF5A2 plays a unique role in protein synthesis and proliferation (Fig 8B). The mechanisms by which *EheIF5A2* gene silencing causes upregulation of *EheIF5A1* gene expression remains elusive. Genetic compensation has been documented in a number of model organisms [62]. It has been proposed in higher eukaryotes that mRNA surveillance pathways, small non-coding RNAs (ncRNAs), upstream open reading frames (uORFs), RNA-binding proteins (RBPs), and micro-RNAs (miRNAs) can potentially be involved in the compensatory response [62].

In conclusion, we have demonstrated that eIF5A and its PTMs are essential for proliferation and might play an important role in differentiation of *Entamoeba*. Furthermore, we have shown that two isoforms of EhDHS play catalytic and regulatory roles in eIF5A modifications. Our results demonstrated for the first time a clear reason for the significance of spermidine, the substrate for hypusination, in *Entamoeba* biology. Our present data should provide a rationale for the PTM of translational machinery and the biosynthetic pathway of polyamines as good targets for the development of new drugs against *Entamoeba*. The specific roles of the unique modifications on EheIF5A1, the identification of DOHH, and the enzymes involved in polyamine biosynthesis need to be elucidated in the future.

## Materials and methods

### Chemicals and reagents

NAD^+^, spermidine, and 1,3-diaminopropane were purchased from Sigma–Aldrich (Tokyo, Japan). Ni^2+^-NTA agarose was purchased from Novagen (Darmstadt, Germany). Lipofectamine and geneticin (G418) were purchased from Invitrogen (Carlsbad, CA, USA). All other chemicals of analytical grade were purchased from Wako (Tokyo, Japan) unless otherwise stated.

### Microorganisms and cultivation

Trophozoites of *E*. *histolytica* clonal strain HM-1:IMSS cl6 and G3 strain [27] were maintained axenically in Diamond’s BI-S-33 medium at 35.5°C as described previously [63]. Trophozoites were harvested in the late-logarithmic growth phase for 2–3 days after inoculation of one-thirtieth to one-twelfth of the total culture volume. After the cultures were chilled on ice for 5 min, trophozoites were collected by centrifugation at 500 g for 10 min at 4°C and washed twice with ice-cold PBS, pH 7.4. *Escherichia coli* BL21 (DE3) strain was purchased from Invitrogen.

### Alignment of DHS and eIF5A protein sequences

Amino acid sequences of DHS protein from *E*. *histolytica* and other organisms were aligned to examine sequences surrounding the key lysine reside. Similarly, amino acid sequences of eIF5A protein from *E*. *histolytica* and human were aligned to examine the key lysine residue that is post translationally modified by hypusination and acetylation. Multiple sequence alignments were generated using CLUSTAL W program (http://clustalw.ddbj.nig.ac.jp/) [64].

### Construction of plasmids for the production of recombinant *E*. *histolytica* DHS1, DHS2, eIF5A1, and eIF5A2

Standard techniques were used for cloning and plasmid construction, essentially as previously described [65]. A DNA fragment corresponding to cDNA encoding *Eh*DHS1, *Eh*DHS2, *Eh*eIF5A1, and *Eh*eIF5A2 was amplified by PCR from *E*. *histolytica* cDNA using the oligonucleotide primers listed in S1 Table to produce a fusion protein containing a histidine-tag (provided by the vector) at the amino terminus. PCR was performed with platinum *pfx* DNA polymerase (Invitrogen) using the following parameters: an initial incubation at 94°C for 2 min; followed by the 30 cycles of denaturation at 94°C for 15 sec; annealing at 50, 45, or 55°C for 30 sec; and elongation at 68°C for 2 min; and a final extension at 68°C for 10 min. The PCR fragments were digested with *Bam*HI/*Hin*dIII (EhDHS1), *Sac*I/*Hin*dIII (EhDHS2) and *Bam*HI and *Sal*I (EheIF5A1 and EheIF5A2), electrophoresed, purified with Gene clean kit II (BIO 101, Vista, CA, USA), and ligated into *Bam*HI/*Hin*dIII, *Sac*I/*Hin*dIII digested pETDuet (Novagen) and *Bam*HI and *Sal*I digested pCOLD-1 (Novagen) in the same orientation as the T7 promoter to produce pETDuet-EhDHS1, pETDuet-EhDHS2, pCOLD-EheIF5A1, and pCOLD-EheIF5A2, respectively. Nucleotide sequences of cloned EhDHS1, DHS2, eIF5A1, and eIF5A2 were verified by sequencing to be identical to the putative protein coding region in the genome database (http://amoebadb.org/amoeba/).

### Construction of a plasmid for co-expression of EhDHS1 and EhDHS2

To construct a plasmid for EhDHS1 and EhDHS2 co-expression, *EhDHS1* and *EhDHS2* genes were amplified using PCR. The upstream and downstream oligonucleotide primers of *EhDHS1* gene, listed in S1 Table, contained *Bam*HI and *Hin*dIII restriction sites. The PCR fragment was subsequently cloned into the *Bam*HI-*Hin*dIII restriction sites of pETDuet (Novagen) to generate the recombinant plasmid pETDuet-DHS1. The His-Tag sequence in pETDuet was fused with *EhDHS1* gene at the N-terminus. The upstream and downstream oligonucleotide primers of *EhDHS2* gene, listed in S1 Table, contained *Aat*II and *Xho*I restriction sites. The stop codon of *EhDHS2* gene was removed so that *EhDHS2* gene is fused with the region encoding the C-terminal S-Tag in pETDuet. The PCR fragment was subsequently cloned into the *Aat*II-*Xho*I restriction sites of pETDuet-*Eh*DHS1 to generate pETDuet-*Eh*DHS1/2.

### Site directed mutagenesis of EhDHS1 and EhDHS2

One-step site directed mutagenesis was conducted on *EhDHS1* and *EhDHS2* gene by QuikChange Site-Directed Mutagenesis Kit (Agilent Technologies). Overlapping primers for site-directed mutagenesis were designed to replace phenylalanine at amino acid 302 of the pETDuet-EhDHS2 plasmid with lysine. Similarly, lysine at amino acid 295 of the pETDuet-EhDHS1 plasmid was replaced with phenylalanine. The primer sets used are listed in S1 Table. PCR reaction was carried out using 100 ng/μl each of the primers, 200 mM dNTPs, 2U of *PfuTurbo* DNA polymerase (Vendor, City, Country) and pETDuet-EhDHS1 or pETDuet-EhDHS2 as a template. The PCR parameters were: 95°C for 30 sec; 16 cycles of 95°C for 30 sec, 55°C for 1 min and 68°C for 10 min. The PCR amplification products were evaluated by agarose gel electrophoresis. PCR products were digested with 1 μl of the *Dpn* I restriction enzyme (10 U/μl) at 37°C for 1 hour to digest the parental (i.e., the non-mutated) supercoiled dsDNA. Approximately 1 μl of the *Dpn* I-treated DNA from each sample reaction was transferred to a tube containing *E*. *coli* XL1-Blue cells. The tube was incubated for 45 seconds at 42°C, and then placed on ice for 2 minutes. Cells were spread onto an LB plate containing 100 μg/ml ampicillin. Bacterial clones containing the plasmid that encodes mutated EhDHS1 or EhDHS2 (DHS1_K295F, DHS2_F302K, respectively) that possesses the intended amino acid mutation (phenylalanine at amino acid 295 in pETDuet-EhDHS1 and lysine at amino acid 302 in pETDuet-EhDHS2) were selected by restriction enzyme digestion and DNA sequencing of the plasmids.

### Bacterial expression and purification of recombinant EhDHS1, EhDHS2, EhDHS1_K295F, EhDHS2_F302K, EheIF5A1, and EheIF5A2

The above-mentioned plasmids were introduced into *E*. *coli* BL21 (DE3) cells by heat shock at 42°C for 1 min. *E*. *coli* BL21 (DE3) strain harboring pCOLD-EheIF5A1, pCOLD-EheIF5A2, pETDuet-EhDHS1, pETDuet-EhDHS2, pETDuetEhDHS1_K295F, pETDuetEhDHS2_F302K, or pETDuet-EhDHS1/2 was grown at 37°C in 100 ml of Luria Bertani medium in the presence of 50 μg/ml ampicillin. The overnight culture was used to inoculate 500 ml of fresh medium, and the culture was further continued at 37°C with shaking at 180 rpm. When A_600_ reached 0.6, 1 mM of isopropyl β-d-thiogalactopyranoside was added, and cultivation was continued for another 24 hr at 15°C except for pETDuet-EhDHS1, pETDuet-EhDHS2, pETDuetEhDHS1_K295F, pETDuetEhDHS2_F302K, pETDuet-EhDHS1/2 which were cultivated for another 6 hr at 30°C. *E*. *coli* cells from the induced culture were harvested by centrifugation at 4,000 g for 20 min at 4°C. The cell pellet was washed with PBS, pH 7.4, re-suspended in 20 ml of the lysis buffer (50 mM Tris–HCl, pH 8.0, 300 mM NaCl, and 10 mM imidazole) containing 0.1% Triton X100 (v/v), 100 μg/ml lysozyme, and 1 mM phenylmethyl sulfonyl fluoride, and incubated at room temperature for 30 min, sonicated on ice and centrifuged at 25,000 g for 15 min at 4°C. The supernatant was mixed with 1.2 ml of 50% Ni^2+^-NTA His-bind slurry, incubated for 1 hr at 4°C with mild shaking. The resin in a column was washed three times with buffer A [50 mM Tris-HCl, pH 8.0, 300 mM NaCl, and 0.1% Triton X-100, v/v] containing 10–50 mM of imidazole. Bound proteins were eluted with buffer A containing 100–300 mM imidazole to obtain recombinant EhDHS1, EhDHS2, a combination of EhDHS1 and 2, EhDHS1_K295F, EhDHS2_F302K, EheIF5A1, and EheIF5A2. After the integrity and the purity of recombinants protein were confirmed with 12% SDS-PAGE analysis, followed by Coomassie Brilliant Blue staining, they were extensively dialyzed twice against the 300-fold volume of 50 mM Tris-HCl, 150 mM NaCl, pH 8.0 containing 10% glycerol (v/v) and Complete Mini protease inhibitor cocktail (Roche, Mannheim, Germany) for 18 hr at 4°C. The dialyzed proteins were stored at –80°C with 30% glycerol in small aliquots until further use. Protein concentrations were spectrophotometrically determined by the Bradford method using bovine serum albumin as a standard as previously described [66].

### Enzyme assays

DHS enzymatic activity was determined by the method described previously [23], unless otherwise stated. The duration of the reaction, buffer pH, and the concentrations of the enzyme and substrates were optimized for the assay. Briefly, a reaction mixture of 50 μl containing 0.2 M glycine-NaOH buffer, pH 9.2, 1 mM dithiothreitol, 1 mM NAD^+^, 1 mM spermidine, 5 μM eIF5A, and the 3 μg of recombinant EhDHS1 or 2 was incubated at 37°C for 60 min and reaction was terminated by the addition of ice cold 10% trichloroacetic acid. The samples were then centrifuged at 10,000 g for 5 min at 4°C and the clear supernatant was analyzed for 1,3 diaminopropane production using reverse phase high performance liquid chromatography as described previously [67].

### Blue Native Polyacrylamide Gel Electrophoresis (BN-PAGE)

Approximately 10 μg of recombinant EhDHS1/2 were electrophoresed on a 4–16% BN-PAGE gel. BN-PAGE was performed using the NativePAGE Novex Bis-Tris Gel System (Invitrogen) according to the manufacturer’s protocol. After electrophoresis, the gel was fixed and destained. An identical gel was also electroblotted onto a polyvinylidene fluoride (PVDF) membrane at 10 V for 2 hr. Following electroblotting, the PVDF membrane was fixed in 10% acetic acid for 20 min, rinsed with deionized water, and subjected to immunoblot analysis as described below.

### Generation of *E*. *histolytica* transformants overexpressing epitope-tagged EhDHS1, EhDHS2, EheIF5A1, EheIF5A2

The protein coding region of *EhDHS1/2* and *EheIF5A1/2* genes were amplified from cDNA by PCR using sense and antisense oligonucleotide primers that contain *Sma*I and *Xho*I sites at the 5’ end, listed in S1 Table. The PCR-amplified DNA fragment was digested with *Sma*I and *Xho*I, and ligated into *Sma*I- and *Xho*I-double digested pEhExHA [68] to produce pEhExHA-DHS1, DHS2, eIF5A1, and eIF5A2. Wild-type trophozoites were transformed with pEhExHA-EhDHS1, pEhExHA-EhDHS2, pEhExHA-EheIF5A1, and pEhExHA-EheIF5A2 by liposome-mediated transfection as previously described [69]. Transformants were initially selected in the presence of 3 μg/ml of geneticin. The geneticin concentration was gradually increased to 6–10 μg/ml during next 2 weeks before the transformants were subjected to analyses.

### Mass spectrometric analysis

To examine whether EheIF5A is hypusinated, we performed immunoprecipitation, followed by mass spectrometry, as described previously [70]. The total mixture of the immunoprecipitated eluates using the lysate from HA-EheIF5A1 and HA-EheIF5A2 expressing transformants were electrophoresed on SDS–PAGE and either visualized by silver staining or reacted with anti-HA antibody by immunoblot analysis. The silver–stained bands corresponding to the predicted molecular weight of EheIF5A1/2 were excised from the gel and subjected to LC–MS/MS analysis, performed at W. M. Keck Biomedical Mass Spectrometry Laboratory, University of Virginia, USA. The detailed methods were previously described [70]. The data were analyzed by database searching using the Sequest search algorithm against Uniprot *E*. *histolytica* eIF5A protein sequences.

### Production of *EhDHS1/2* and *EheIF5A1/A2* gene-silenced strains

In order to construct plasmids for small antisense RNA-mediated transcriptional gene silencing [27] of *EhDHS1/2* and *EheIF5A1/A2* genes, a fragment corresponding to a 420-bp long fragment corresponding to the amino-terminal portion of the entire protein was amplified by PCR from cDNA using sense and antisense oligonucleotide primers containing *Stu*I and *Sac*I restriction sites as shown in S1 Table. The PCR-amplified product was digested with *Stu*I and *Sac*I, and ligated into the *Stu*I- and *Sac*I-double digested psAP2-Gunma [33] to construct gene silencing plasmids psAP2G-DHS1, psAP2G-DHS2, psAP2G-eIF5A1 and psAP2G-eIF5A2. The trophozoites of G3 strain were transformed with either empty vector or silencing plasmid by liposome-mediated transfection as previously described [69]. Transformants were initially selected in the presence of 1 μg/ml geneticin, and the geneticin concentrations were gradually increased to 6–10 μg/ml during next two weeks prior to subjecting the transformants to analyses.

### Growth assay of *E*. *histolytica* trophozoites

Approximately 6×10^4^ exponentially growing trophozoites of *E*. *histolytica* G3 strain transformed with psAP2G-DHS2, psAP2G-eIF5A2, and psAP2G (control) were inoculated into 6 ml of fresh BI-S-33 medium containing 10 μg/ml geneticin, and the parasites were counted every 24 hr on a haemocytometer.

### Surface sensing of translation (SUnSET)

SUnSET analysis was conducted to assess translational machinery in *E*. *histolytica* as previously described [34]. Approximately 2×10^6^ trophozoites were incubated with 10 μg/ml puromycin (Sigma-Aldrich) for 15 min before or after incubation with 100 μg/ml cycloheximide for 10 min at 37°C. After cells were collected by centrifugation, proteins were precipitated by incubating with 20% (v/v) TCA on ice for 10 min, followed by centrifugation at 2,200 g for 5 min and washing the pellet with 5% (v/v) TCA. The protein pellet was resuspended in 2X SDS running buffer and incubated in boiling water for 10 min. The lysate was frozen at –80°C until analyzed via immunoblot analysis (see below). Anti-puromycin mouse monoclonal antibodies (Sigma-Aldrich) were used at a 1:2500 dilution. As a loading control, blots were reacted with anti-cysteine synthase 1 (CS1) rabbit polyclonal antiserum [29] at a 1:1000 dilution.

### *E*. *invadens* culture, encystation, and excystation

Trophozoites of *E*. *invadens* IP-1 strain were cultured axenically in BI-S-33 medium at 26°C. To induce encystation, 2 week-old *E*. *invadens* cultures were passaged to 47% LG medium lacking glucose [13, 37] at approximately 6×10^5^ cells/ml. Amebae were collected at various time points, and the formation of cysts was assessed in triplicate by virtue of the resistance to 0.05% sarkosyl using 0.22% trypan blue to selectively stain dead cells. Cysts were also verified by cyst wall staining by incubating amebae with calcofluor white (fluorescent brightener; Sigma-Aldrich) at room temperature. For excystation, cysts formed as above were harvested at 72 hr and any remaining trophozoites were destroyed by hypotonic lysis by incubating overnight (~16 hr) in distilled water at 4°C. Excystation was induced by incubating 8×10^5^ cysts in 6 ml of LG medium supplemented with 1 mg/ml bile, 40 mM sodium bicarbonate, 1% glucose, 2.8% vitamin mix, and 10% adult bovine serum for 48 hr [71]. Excystation was conducted in duplicates and the efficiency was determined by counting trophozoites on a haemocytometer.

### Cell fractionation and immunoblot analysis

Trophozoites of amoeba transformants expressing pEhExHA-DHS1, pEhExHA-DHS2, pEhExHA-eIF5A1, or pEhExHA-eIF5A2, were washed three times with PBS containing 2% glucose. After resuspension in homogenization buffer (50 mM Tris-HCl, pH 7.5, 250 mM sucrose, 50 mM NaCl and 0.5 mg/ml E-64 protease inhibitor), the cells were disrupted mechanically by a Dounce homogenizer on ice, centrifuged at 500 g for 5 min, and the supernatant was collected to remove unbroken cells. The supernatant fraction was centrifuged at 5,000 g for 10 min to isolate pellet and supernatant fractions. The 5,000 g supernatant fraction was further centrifuged at 100,000 g for 60 min to produce a 100,000 g supernatant and pellet fractions. The pellets at each step were further washed twice with homogenization buffer and re-centrifuged at 100,000 g for 10 min to minimize carryover.

Cell lysates and culture supernatants were separated on 12–15% (w/v) SDS-PAGE and subsequently electro transferred onto nitrocellulose membranes (Hybond-C Extra; Amersham Biosciences UK, Little Chalfont, Bucks, UK) as previously described [72]. Non-specific binding was blocked by incubating the membranes for 1.5 hr at room temperature in 5% non-fat dried milk in TBST (50 mM Tris-HCl, pH 8.0, 150 mM NaCl and 0.05% Tween-20). The blots were reacted with anti-HA mouse monoclonal (clone 11MO, Covance, Princeton, NJ, USA), anti-hypusine rabbit polyclonal antibodies [28], anti-CS1 [29] and anti-cysteine protease binding family protein 1 (CPBF1) [30] rabbit polyclonal antisera at a dilution of 1:500 to 1:1000. CPBF1 and CS1 served as controls for membrane and cytosolic fractions, respectively. The membranes were washed with TBST and further reacted either with alkaline phosphatase-conjugated anti-mouse or anti-rabbit IgG secondary antibody (1:2000) or with horse radish peroxidase-conjugated anti-mouse or anti-rabbit IgG antisera (1:20,000) (Invitrogen) at room temperature for 1 hr. After washings with TBST, specific proteins were visualized with alkaline phosphatase conjugate substrate kit (Bio-Rad) and images were scanned with Image Scanner (Amersham Pharmacia Biotech, Piscataway, NJ, USA) or the fluorescent signal of each protein was measured with a chemiluminescence detection system (Millipore) using Scion Image software (Scion Corp., Frederick, MD).

## Supporting information

S1 Fig Multiple sequence alignment of DHS protein sequences. Alignment of DHS protein sequences from *E*. *histolytica* [EhDHS1 (XP_653614), EhDHS2 (XP_653426)], *H*. *sapiens* (P49366) and *E*. *invadens* [EiDHS1(XP_004260556), EiDHS2 (XP_004257561)]. The catalytic lysine residue is shown in red background, whereas NAD^+^ and spermidine binding sites are shown in green and yellow background, respectively. The conserved residues are marked by asterisks (*) while similar amino acids are shown either with periods (.) or colons (:). Sequence alignment was performed using ClustalW.
(TIF)

S2 Fig Multiple sequence alignment of amino acid sequences of four entries corresponding to *E*. *histolytica* eIF5A. Accession numbers of these sequences are as follows: EHI_186480 (XP_651531), EHI_151810 (XP_657397), EHI_151540 (XP_657374), and EHI_177460 (XP_655916). The conserved residues are marked by asterisks (*) while similar amino acids are shown either with periods (.) or colons (:). The conserved lysine residue, which was supposed to be hypusinated, is highlighted in red. Sequence alignment was performed using ClustalW.
(TIF)

S3 Fig **(A) Purification of recombinant EhDHS1, EhDHS2, EheIF5A1, and EheIF5A2.** Protein samples at each step of purification were subjected to 15% SDS-PAGE under reducing conditions, and then stained with Coomassie Brilliant Blue R250. **(B)** Purification of the recombinant EhDHS mutants.
(TIF)

S4 Fig Immunoblot analysis of *E*. *histolytica* transformant strains expressing HA-tagged EhDHS1, EhDHS2, EheIF5A1 and EheI5A2. Approximately 40 μg of total lysates were electrophoresed on a SDS-PAGE gel under reducing conditions and subjected to immunoblot analysis using anti-HA and anti-hypusine antibodies.
(TIF)

S5 Fig In vitro deoxyhypusination of EheIF5A by co-expressed recombinant EhDHS1 and 2, and verification of the specificity of anti-hypusine antibody. Recombinant EheIF5A1 or EheIF5A2 was incubated with 1 mM spermidine, 0.5 mM NAD^+^, 3μg of co-expressed recombinant EhDHS1 and 2 (N-terminal His-tag EhDHS1 and C-terminal S-tag EhDHS2), and the mixtures were subjected to SDS-PAGE and immunoblot analyses using anti-hypusine, anti-His, and anti-S tag antibodies.
(TIF)

S6 Fig Validation of expression of endogenous and epitope tagged EheIF5A isoforms in *E*. *histolytica* trophozoites. Approximately 40 μg of total lysate from the transformant expressing HA-EheIF5A1 and HA-EheIF5A2 and HA was electrophoresed on a SDS-PAGE gel under reducing conditions and subjected to immunoblot analysis using anti-EheIF5A1, anti-EheIF5A2 and anti-HA antibodies.
(TIF)

S7 Fig **(A)** Percent amino acid identity among *E*. *histolytica* and *E*. *invadens* eIF5A isoforms by ClustalW multiple sequence alignment score. GenBank accession numbers: EheIF5A1 (XP_657374), EheIF5A2 (XP_651531), EieIF5A1 (XP_004255257), EieIF5A2 (XP_004258351), EieIF5A3 (XP_004260381). **(B)** Amino acid sequence alignment of *E*. *histolytica*, *E*. *invadens* and *Homo sapiens* eIF5A isoforms. Accession numbers of these sequences are as follows: EheIF5A1 (XP_657374); EheIF5A2 (XP_651531); EieIF5A1 (XP_004255257), EieIF5A2 (XP_004258351), EieIF5A3 (XP_004260381), HseIF5A1 (P63241); and HseIF5A2 (AAG23176). The conserved residues are marked by asterisks (*) while similar amino acids are shown either with periods (.) or colons (:). The conserved lysine residue, which was supposed to be hypusinated, is highlighted in red. Sequence alignment was performed using ClustalW.
(TIF)

S8 Fig Phylogenetic tree of eIF5A from *Entamoeba* and other species. Maximum likelihood tree inferred by MEGA X program with JTT matrix-based model is shown. 132 unambiguously aligned positions from 39 sequences were used for the analysis. Branch lengths are proportional to estimated numbers of substitutions. Bootstrap proportion (BP) values (shown in percentage) are shown on the internal branches. Species names and accession numbers of the sequences are also indicated.
(TIF)

S9 Fig Immunoblot analysis of *E*. *invadens* trophozoites. Approximately 30 μg of total lysates was electrophoresed on a 15% SDS-PAGE gel under reducing conditions and subjected to immunoblot analysis using anti-hypusine, anti-EheIF5A1 and anti-EheIF5A2 antibodies.
(TIF)

S10 Fig The levels of steady state mRNA expression of *EheIF5A* isoforms during encystation. **(A)** Kinetics of encystation. The percentages of the amoebae resistant to 0.05% sarkosyl during encystation. **(B)** The steady-state levels of transcripts of *EieIF5A1*, *EieI5FA2*, *EieIF5A1*, and *EiActin* genes measured by semi-quantitative RT-PCR during encystation. cDNA from different time points during encystation was subjected to 30 cycles of PCR using specific primers for the *EheIF5A1*, *EheI5FA2*, *EheIF5A3* and *Ehactin* genes. *Ehactin* gene served as a control. PCR analysis of samples without reverse transcription was also used to exclude the possibility of genomic DNA contamination.
(TIF)

S11 Fig **(A)** Representative immunofluorescence assay (IFA) micrographs of HA-eIF5A1 and HA-eIF5A2 expressed in *E*. *histolytica* trophozoites, double stained with anti-HA antibody (green) and anti-CS1 antiserum (red) respectively. EhCS1 (Cysteine synthase 1) served as a cytosolic control. Scale bar, 10 μm. **(B)** eIF5A2gs (only eIF5A1 expressed) and control (pSAP2G; only eIF5A2 expressed).
(TIF)

S1 Table List of primers used in this study. (XLSX)
