# Supplementary figures and images for: Eukaryotic translation initiation factor 5A and its posttranslational modifications play an important role in proliferation and potentially in differentiation of the human enteric protozoan parasite Entamoeba histolytica
Source: PLoS Pathog. 2021 Feb 16;17(2):e1008909. doi: 10.1371/journal.ppat.1008909 (PMC7909649; doi:10.1371/journal.ppat.1008909)

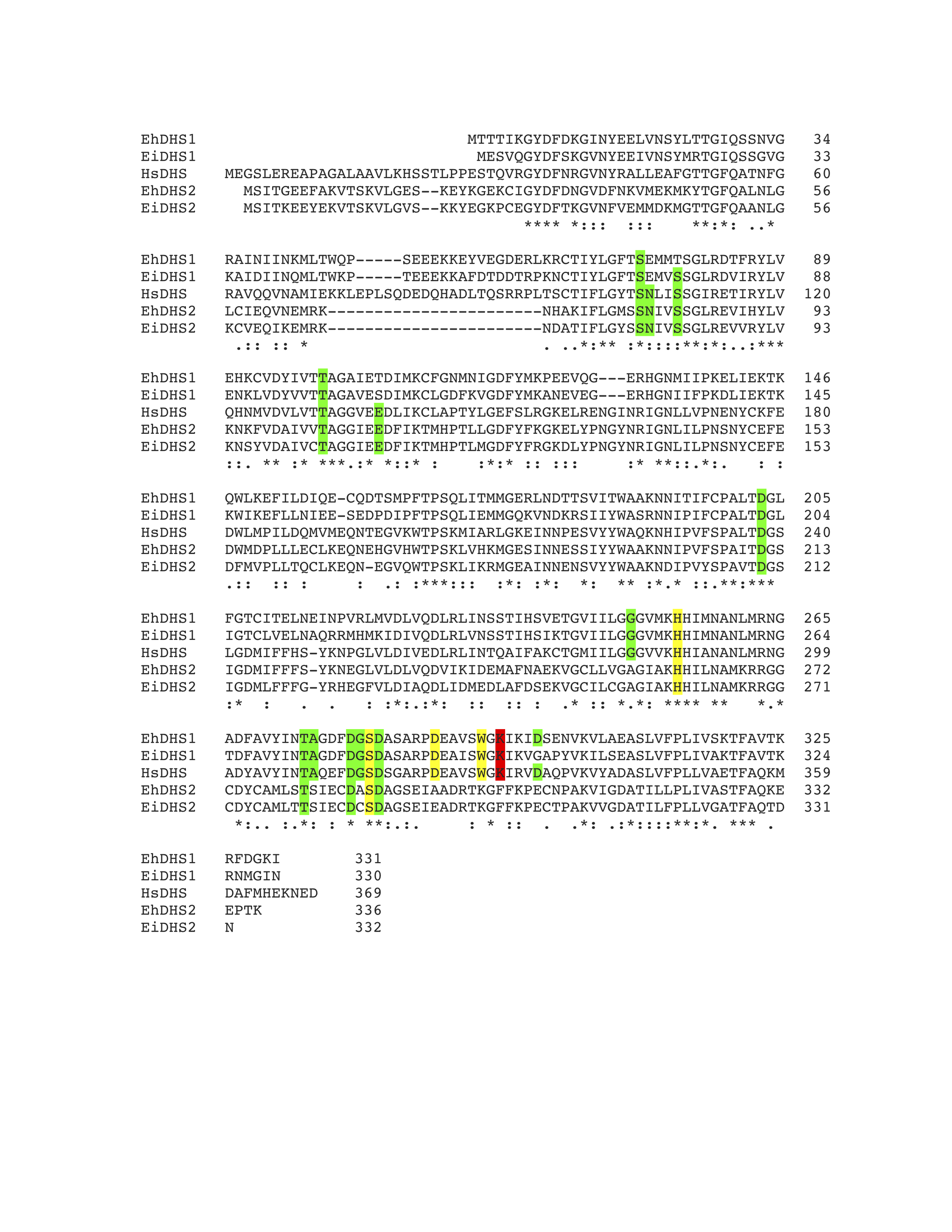

Supplement: S1 Fig — Alignment of DHS protein sequences from E. histolytica [EhDHS1 (XP_653614), EhDHS2 (XP_653426)], H. sapiens (P49366) and E. invadens [EiDHS1(XP_004260556), EiDHS2 (XP_004257561)]. The catalytic lysine residue is shown in red background, whereas NAD+ and spermidine binding sites are shown in green and yellow background, respectively. The conserved residues are marked by asterisks (*) while similar amino acids are shown either with periods (.) or colons (:). Sequence alignment was performed using ClustalW. (TIF) [file ppat.1008909.s001.tif]

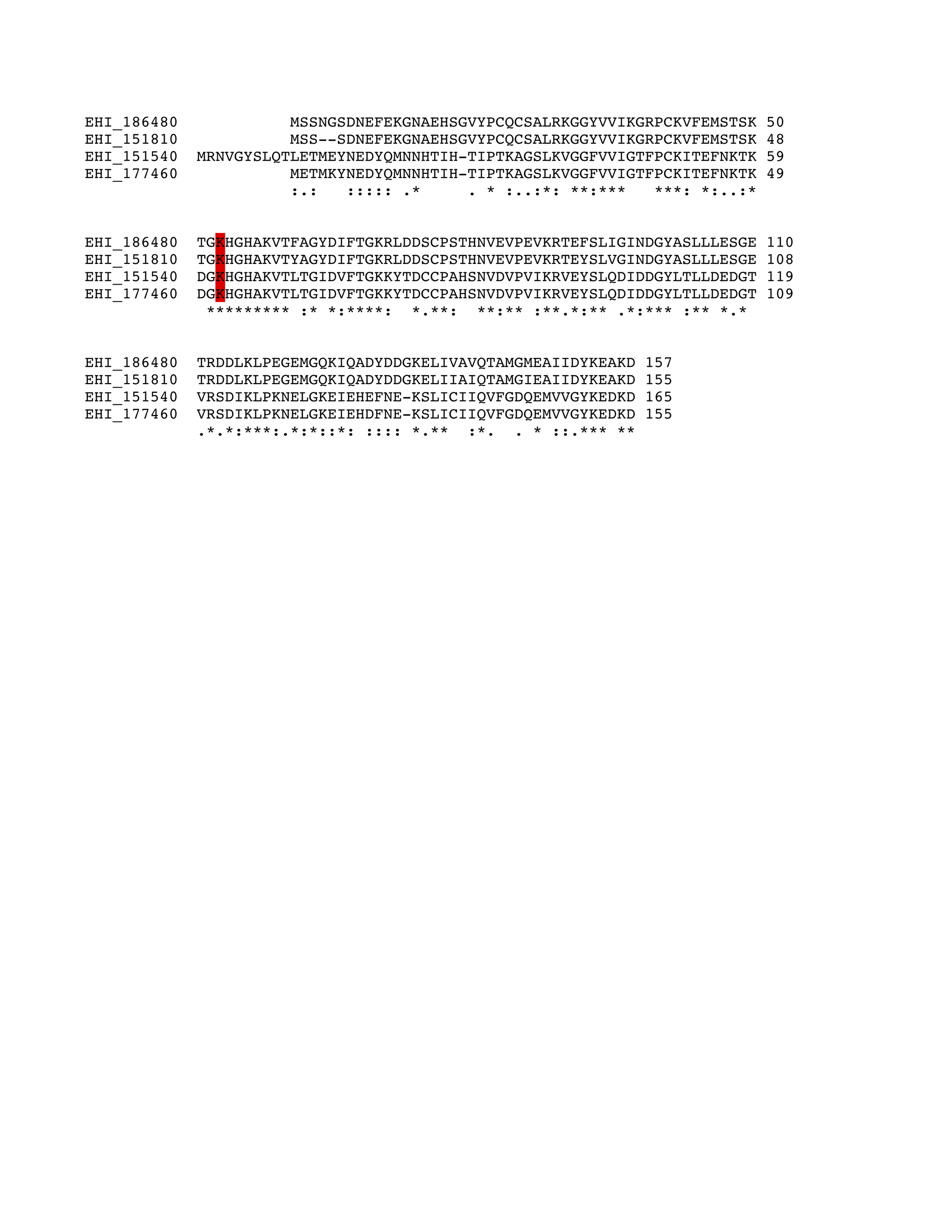

Supplement: S2 Fig — Accession numbers of these sequences are as follows: EHI_186480 (XP_651531), EHI_151810 (XP_657397), EHI_151540 (XP_657374), and EHI_177460 (XP_655916). The conserved residues are marked by asterisks (*) while similar amino acids are shown either with periods (.) or colons (:). The conserved lysine residue, which was supposed to be hypusinated, is highlighted in red. Sequence alignment was performed using ClustalW. (TIF) [file ppat.1008909.s002.tif]

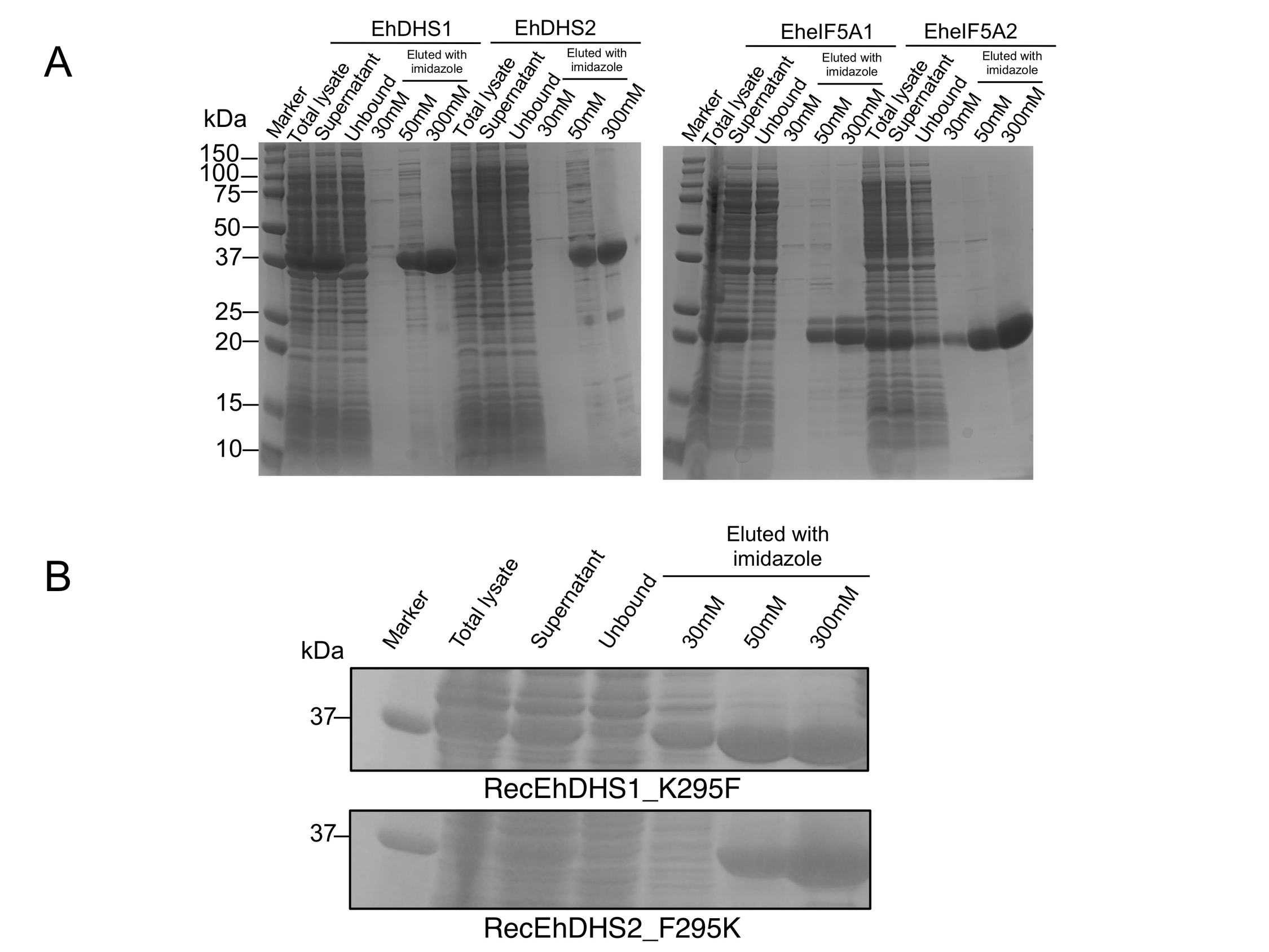

Supplement: S3 Fig — (A) Purification of recombinant EhDHS1, EhDHS2, EheIF5A1, and EheIF5A2. Protein samples at each step of purification were subjected to 15% SDS-PAGE under reducing conditions, and then stained with Coomassie Brilliant Blue R250. (B) Purification of the recombinant EhDHS mutants. (TIF) [file ppat.1008909.s003.tif]

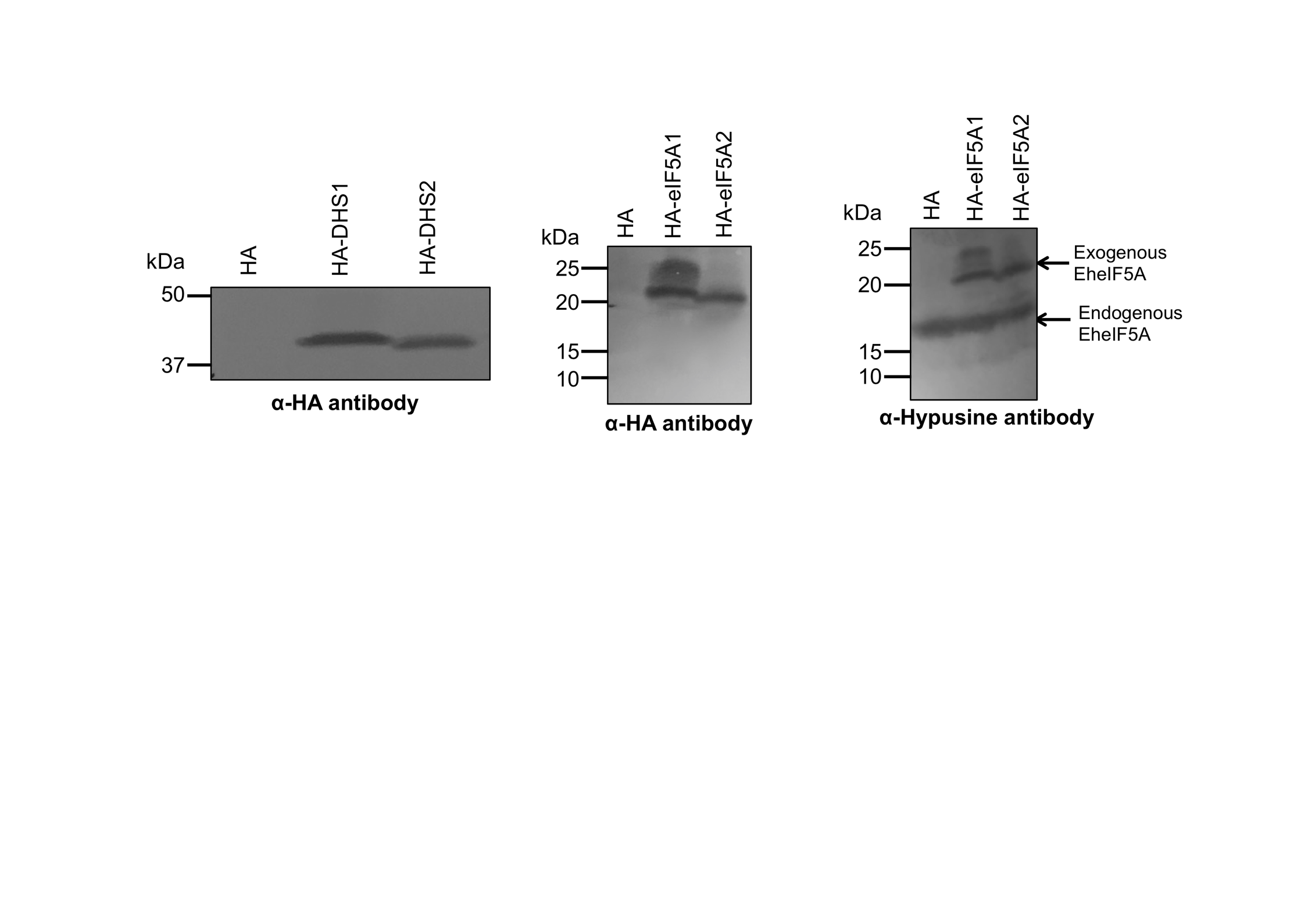

Supplement: S4 Fig — Approximately 40 μg of total lysates were electrophoresed on a SDS-PAGE gel under reducing conditions and subjected to immunoblot analysis using anti-HA and anti-hypusine antibodies. (TIF) [file ppat.1008909.s004.tif]

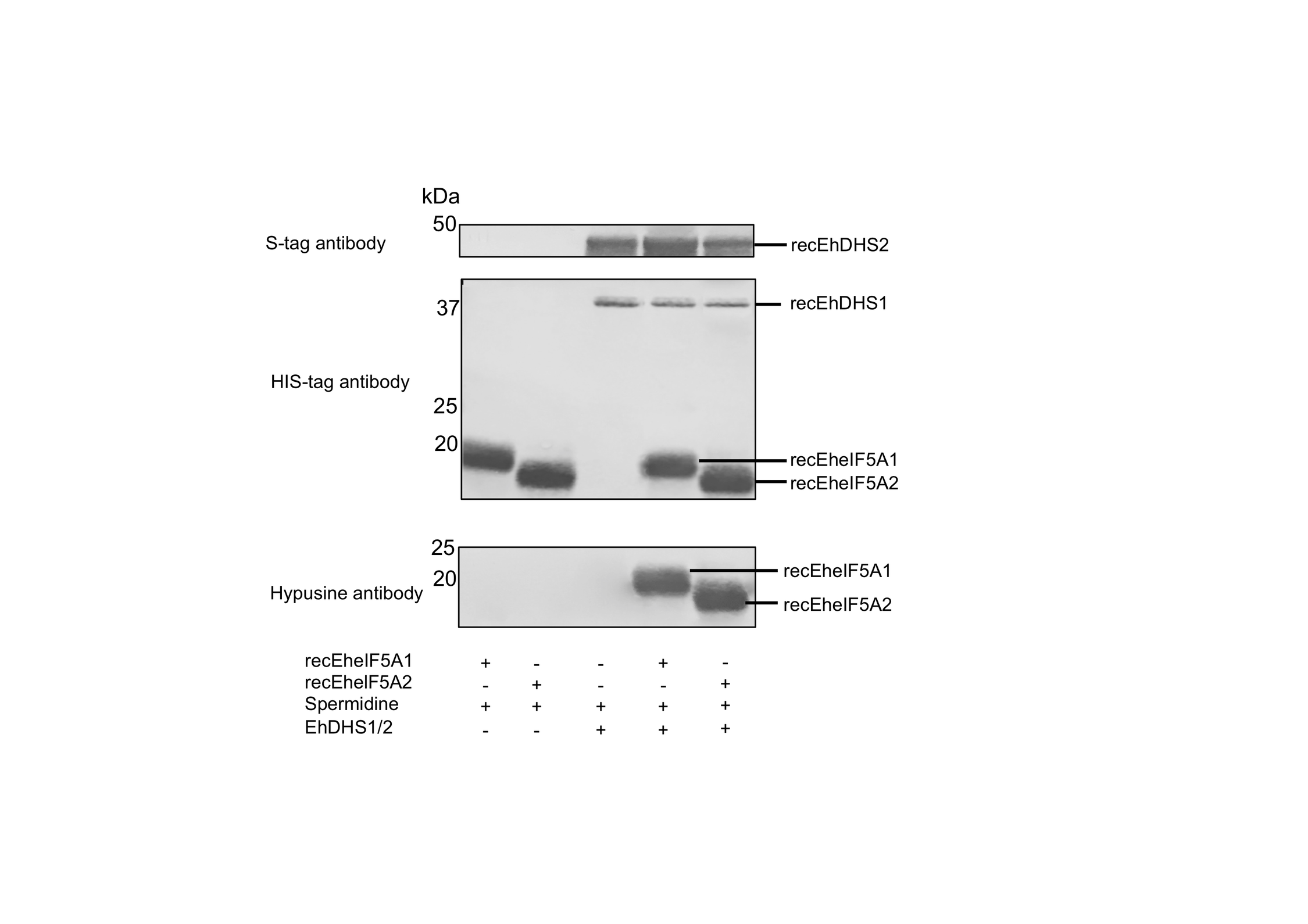

Supplement: S5 Fig — Recombinant EheIF5A1 or EheIF5A2 was incubated with 1 mM spermidine, 0.5 mM NAD+, 3μg of co-expressed recombinant EhDHS1 and 2 (N-terminal His-tag EhDHS1 and C-terminal S-tag EhDHS2), and the mixtures were subjected to SDS-PAGE and immunoblot analyses using anti-hypusine, anti-His, and anti-S tag antibodies. (TIF) [file ppat.1008909.s005.tif]

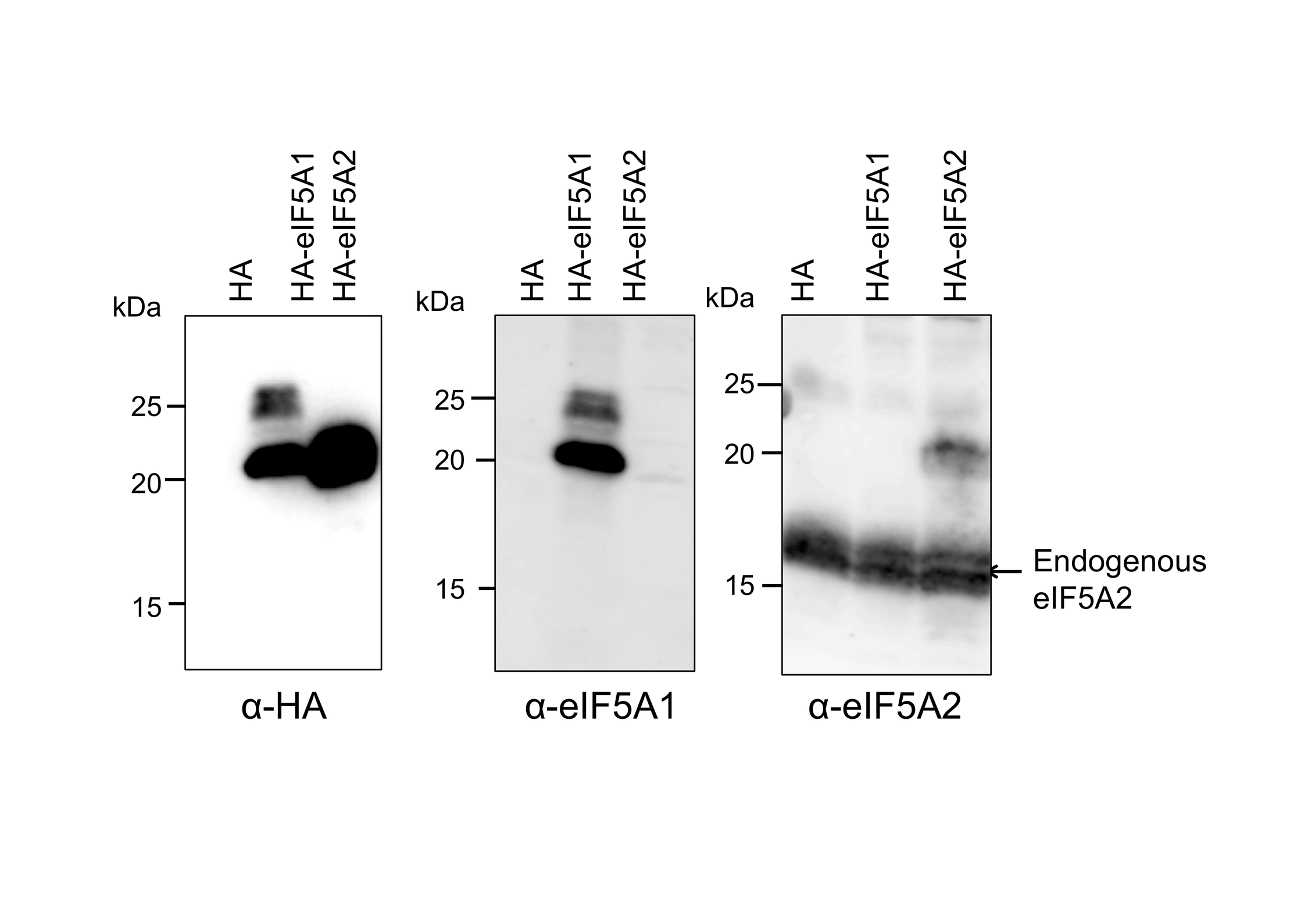

Supplement: S6 Fig — Approximately 40 μg of total lysate from the transformant expressing HA-EheIF5A1 and HA-EheIF5A2 and HA was electrophoresed on a SDS-PAGE gel under reducing conditions and subjected to immunoblot analysis using anti-EheIF5A1, anti-EheIF5A2 and anti-HA antibodies. (TIF) [file ppat.1008909.s006.tif]

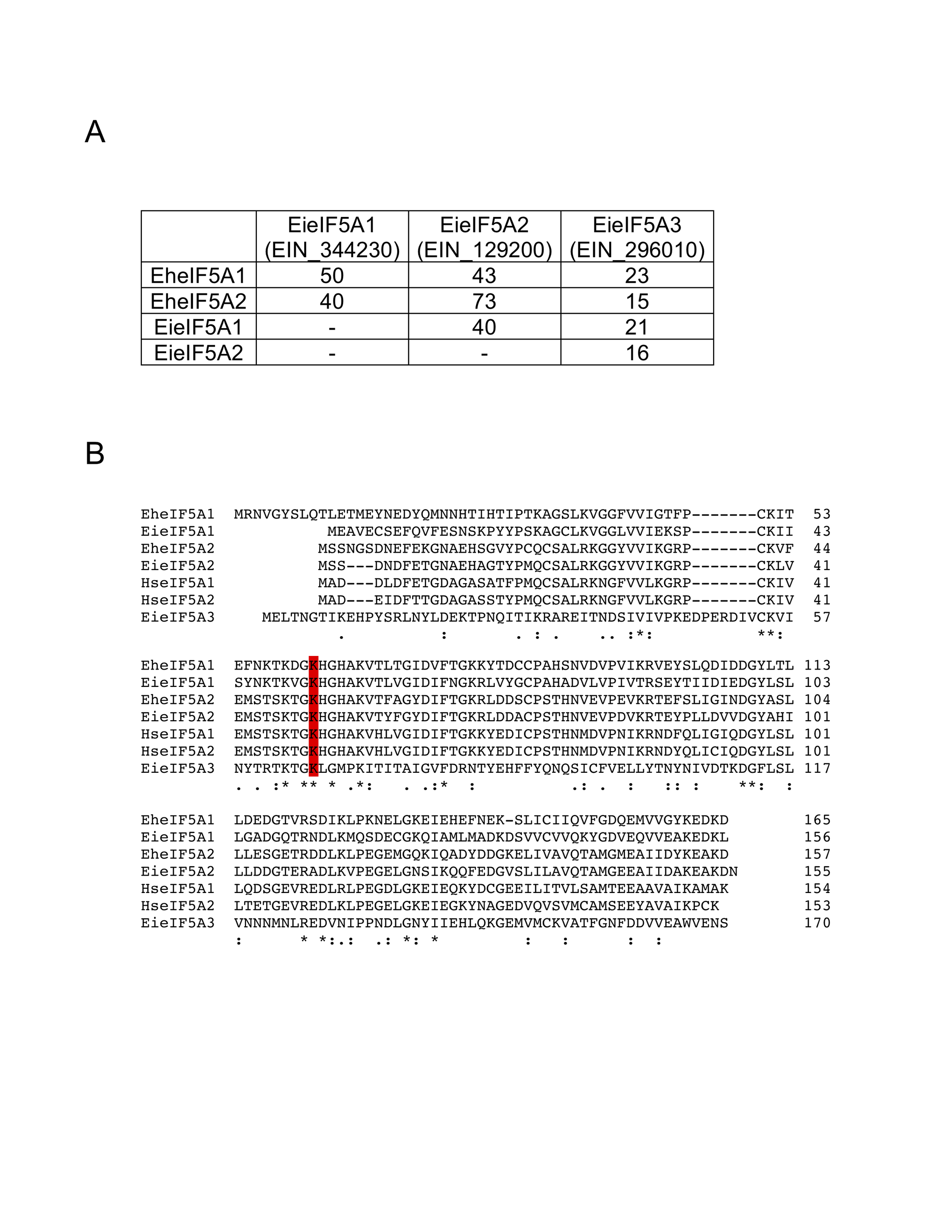

Supplement: S7 Fig — (A) Percent amino acid identity among E. histolytica and E. invadens eIF5A isoforms by ClustalW multiple sequence alignment score. GenBank accession numbers: EheIF5A1 (XP_657374), EheIF5A2 (XP_651531), EieIF5A1 (XP_004255257), EieIF5A2 (XP_004258351), EieIF5A3 (XP_004260381). (B) Amino acid sequence alignment of E. histolytica, E. invadens and Homo sapiens eIF5A isoforms. Accession numbers of these sequences are as follows: EheIF5A1 (XP_657374); EheIF5A2 (XP_651531); EieIF5A1 (XP_004255257), EieIF5A2 (XP_004258351), EieIF5A3 (XP_004260381), HseIF5A1 (P63241); and HseIF5A2 (AAG23176). The conserved residues are marked by asterisks (*) while similar amino acids are shown either with periods (.) or colons (:). The conserved lysine residue, which was supposed to be hypusinated, is highlighted in red. Sequence alignment was performed using ClustalW. (TIF) [file ppat.1008909.s007.tif]

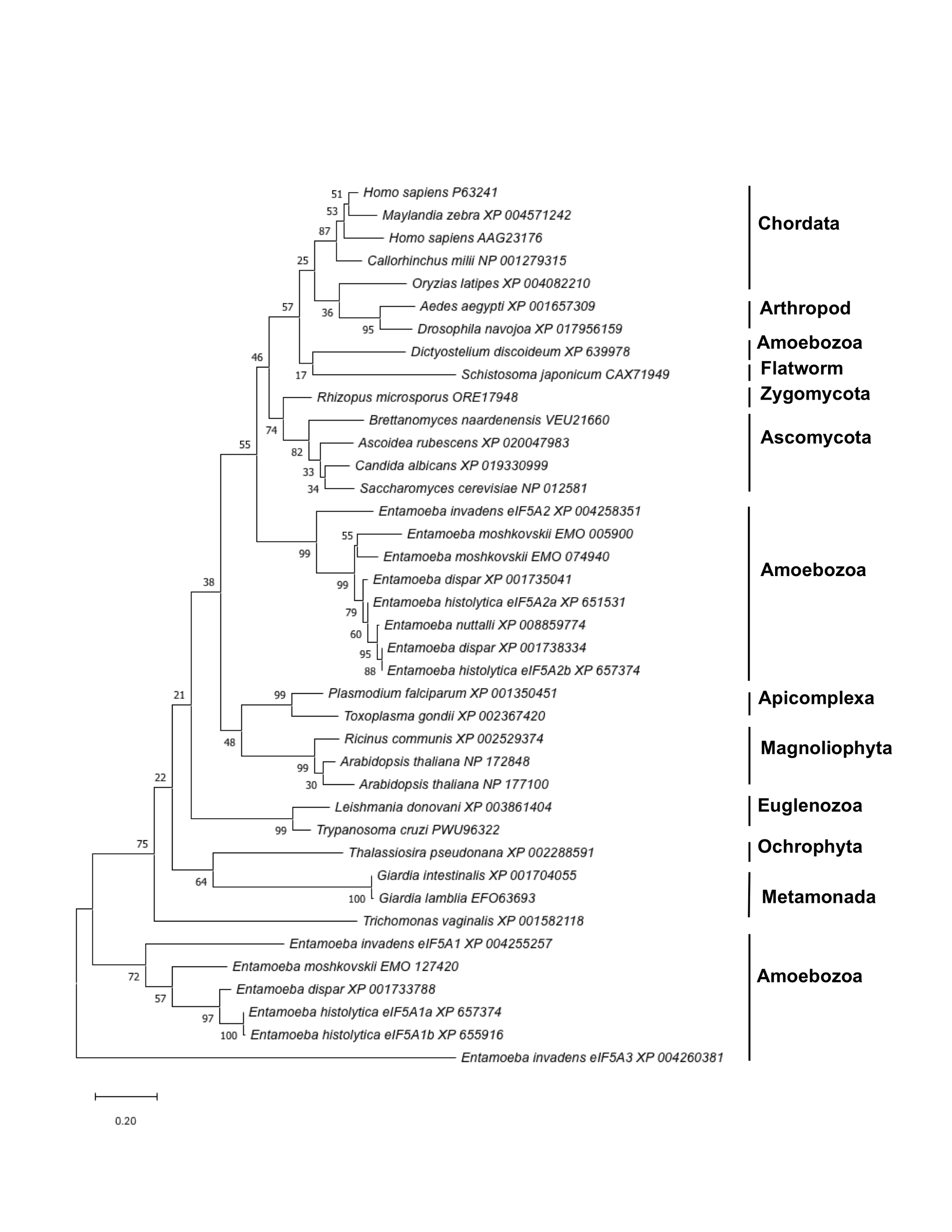

Supplement: S8 Fig — Maximum likelihood tree inferred by MEGA X program with JTT matrix-based model is shown. 132 unambiguously aligned positions from 39 sequences were used for the analysis. Branch lengths are proportional to estimated numbers of substitutions. Bootstrap proportion (BP) values (shown in percentage) are shown on the internal branches. Species names and accession numbers of the sequences are also indicated. (TIF) [file ppat.1008909.s008.tif]

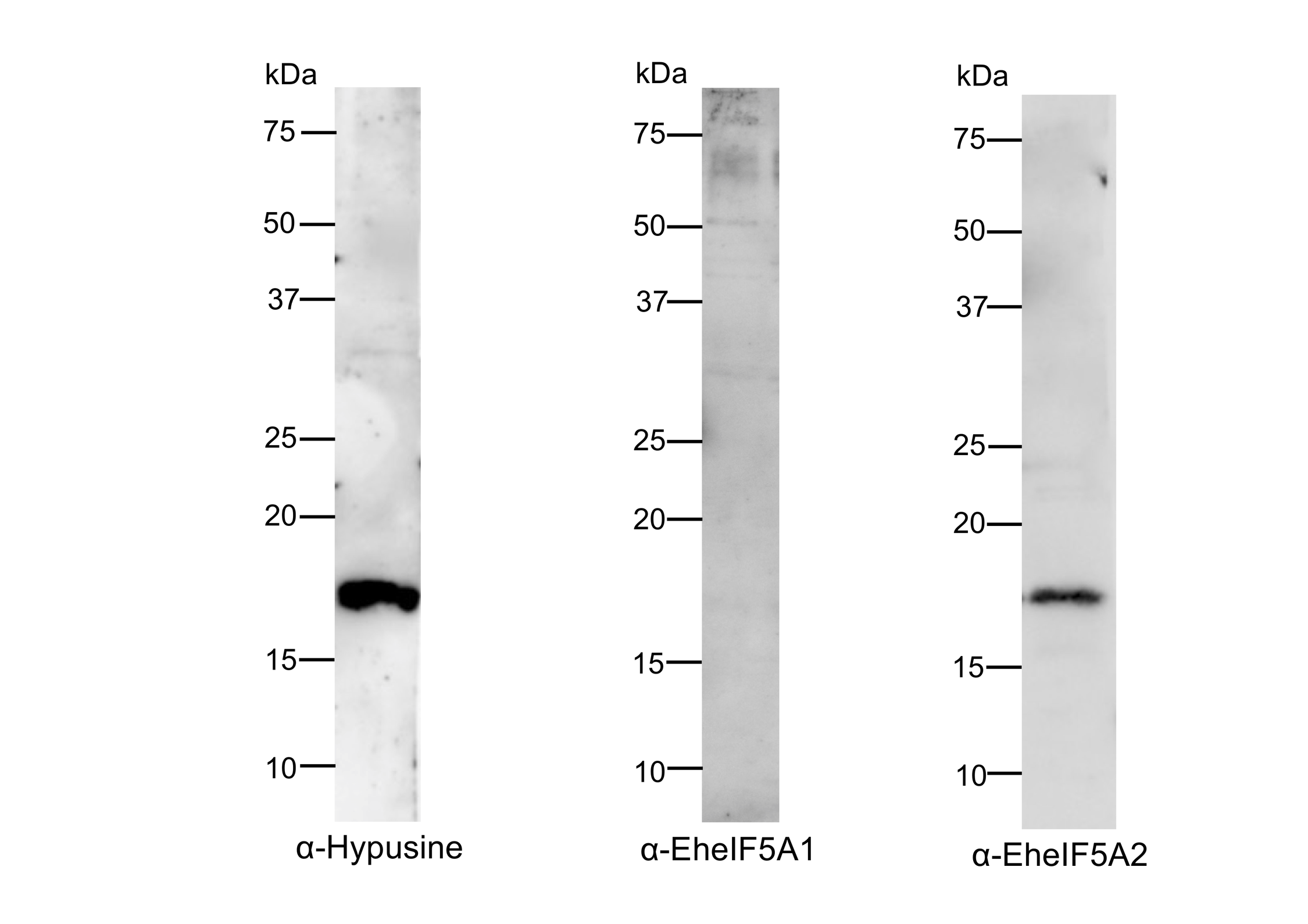

Supplement: S9 Fig — Approximately 30 μg of total lysates was electrophoresed on a 15% SDS-PAGE gel under reducing conditions and subjected to immunoblot analysis using anti-hypusine, anti-EheIF5A1 and anti-EheIF5A2 antibodies. (TIF) [file ppat.1008909.s009.tif]

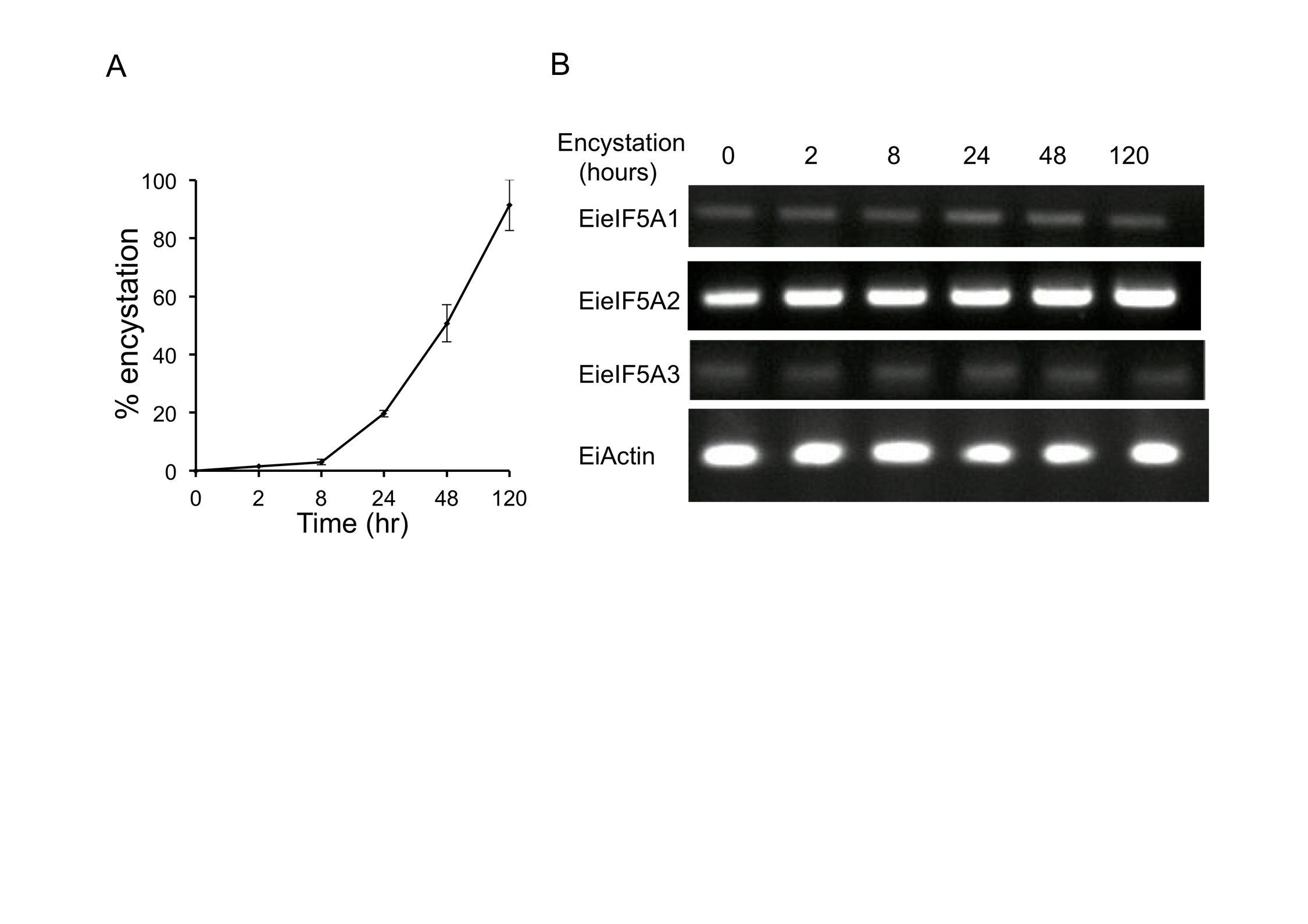

Supplement: S10 Fig — (A) Kinetics of encystation. The percentages of the amoebae resistant to 0.05% sarkosyl during encystation. (B) The steady-state levels of transcripts of EieIF5A1, EieI5FA2, EieIF5A1, and EiActin genes measured by semi-quantitative RT-PCR during encystation. cDNA from different time points during encystation was subjected to 30 cycles of PCR using specific primers for the EheIF5A1, EheI5FA2, EheIF5A3 and Ehactin genes. Ehactin gene served as a control. PCR analysis of samples without reverse transcription was also used to exclude the possibility of genomic DNA contamination. (TIF) [file ppat.1008909.s010.tif]

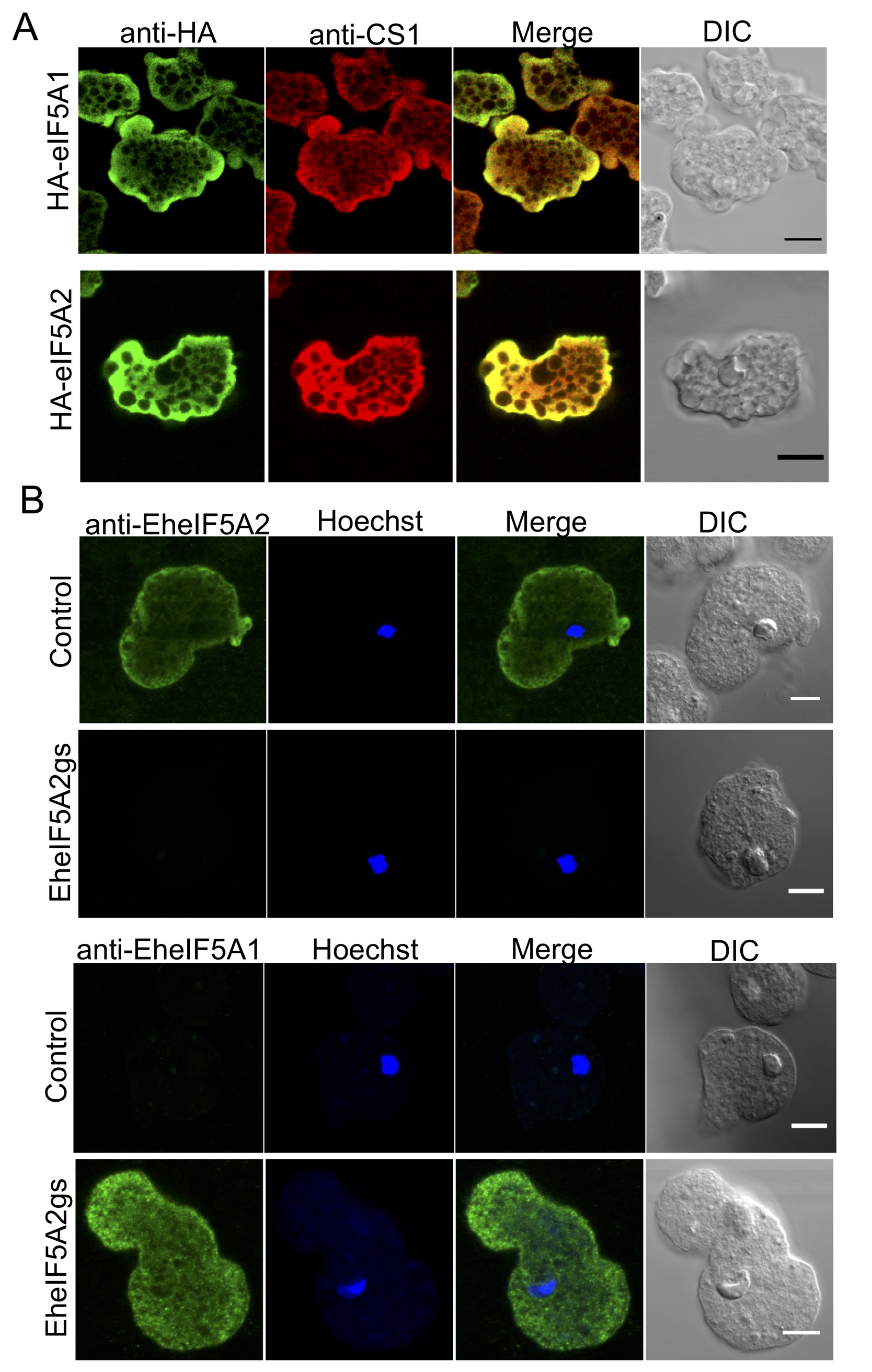

Supplement: S11 Fig — (A) Representative immunofluorescence assay (IFA) micrographs of HA-eIF5A1 and HA-eIF5A2 expressed in E. histolytica trophozoites, double stained with anti-HA antibody (green) and anti-CS1 antiserum (red) respectively. EhCS1 (Cysteine synthase 1) served as a cytosolic control. Scale bar, 10 μm. (B) eIF5A2gs (only eIF5A1 expressed) and control (pSAP2G; only eIF5A2 expressed). (TIF) [file ppat.1008909.s011.tif]
